# Defatting of donor transplant livers during normothermic perfusion—a randomised clinical trial: study protocol for the DeFat study

**DOI:** 10.1186/s13063-024-08189-4

**Published:** 2024-06-17

**Authors:** Syed Hussain Abbas, Carlo D. L. Ceresa, Leanne Hodson, David Nasralla, Christopher J. E. Watson, Hynek Mergental, Constantin Coussios, Fotini Kaloyirou, Kerrie Brusby, Ana Mora, Helen Thomas, Daphne Kounali, Katie Keen, Joerg-Matthias Pollok, Rohit Gaurav, Satheesh Iype, Wayel Jassem, M. Thamara PR Perera, Abdul Rahman Hakeem, Simon Knight, Peter J. Friend

**Affiliations:** 1 Nuffield Department of Surgical Sciences, University of Oxford, The Churchill Hospital, Oxford, OX3 7LJ UK; 2 Royal Free London NHS Foundation Trust, The Royal Free Hospital, Pond St, Hampstead, London, NW3 2QG UK; 3 Oxford Centre for Diabetes, Endocrinology and Metabolism, University of Oxford, The Churchill Hospital, Oxford, OX3 7LJ UK; 4 Department of Surgery, Addenbrooke’s Hospital, Hills Road, University of Cambridge, Box 202, Cambridge, CB2 2QQ UK; 5 Queen Elizabeth Hospital, University Hospitals Birmingham NHS Foundation Trust, Mindelsohn Way, Birmingham, B15 2TH UK; 6 TransMedics Inc, 200 Minuteman Road, Andover, MA 01810 USA; 7 Institute of Biomedical Engineering, Old Road Campus Research Building, University of Oxford, Oxford, OX3 7DQ UK; 8 NHSBT CTU, Long Road, Cambridge, CB2 0PT UK; 9 Victor Phillip Dahdaleh Heart and Lung Research Institute, University of Cambridge, Cambridge Biomedical Campus, Hills Road, Cambridge, CB2 0BB UK; 10 NHS Blood and Transplant Clinical Trials Unit, Fox Den Road, Stoke Gifford, Bristol, BS34 8RR UK; 11 Oxford Clinical Trials Research Unit (OCTRU), Centre for Statistics in Medicine (CSM), Nuffield Department of Orthopaedics, Rheumatology and Musculoskeletal Sciences (NDORMS), Medical Sciences Division, The Botnar Research Centre, University of Oxford, Windmill Road, Oxford, OX3 7LD UK; 12 Kings College Hospital, King’s College Hospital NHS Foundation Trust, Denmark Hill, London, SE5 9RS UK; 13 St James’s University Hospital, Leeds Teaching Hospitals NHS Trust, Beckett Street, Leeds, LS9 7TF UK

**Keywords:** Hepatic steatosis, Non-alcoholic fatty liver disease (NAFLD), Normothermic machine perfusion (NMP), Defatting, Functional assessment, Liver transplantation

## Abstract

**Background:**

Liver disease is the third leading cause of premature death in the UK. Transplantation is the only successful treatment for end-stage liver disease but is limited by a shortage of suitable donor organs. As a result, up to 20% of patients on liver transplant waiting lists die before receiving a transplant. A third of donated livers are not suitable for transplant, often due to steatosis. Hepatic steatosis, which affects 33% of the UK population, is strongly associated with obesity, an increasing problem in the potential donor pool. We have recently tested defatting interventions during normothermic machine perfusion (NMP) in discarded steatotic human livers that were not transplanted. A combination of therapies including forskolin (NKH477) and L-carnitine to defat liver cells and lipoprotein apheresis filtration were investigated. These interventions resulted in functional improvement during perfusion and reduced the intrahepatocellular triglyceride (IHTG) content. We hypothesise that defatting during NMP will allow more steatotic livers to be transplanted with improved outcomes.

**Methods:**

In the proposed multi-centre clinical trial, we will randomly assign 60 livers from donors with a high-risk of hepatic steatosis to either NMP alone or NMP with defatting interventions. We aim to test the safety and feasibility of the defatting intervention and will explore efficacy by comparing ex-situ and post-reperfusion liver function between the groups. The primary endpoint will be the proportion of livers that achieve predefined functional criteria during perfusion which indicate potential suitability for transplantation. These criteria reflect hepatic metabolism and injury and include lactate clearance, perfusate pH, glucose metabolism, bile composition, vascular flows and transaminase levels. Clinical secondary endpoints will include proportion of livers transplanted in the two arms, graft function; cell-free DNA (cfDNA) at follow-up visits; patient and graft survival; hospital and ITU stay; evidence of ischemia-reperfusion injury (IRI); non-anastomotic biliary strictures and recurrence of steatosis (determined on MRI at 6 months).

**Discussion:**

This study explores ex-situ pharmacological optimisation of steatotic donor livers during NMP. If the intervention proves effective, it will allow the safe transplantation of livers that are currently very likely to be discarded, thereby reducing waiting list deaths.

**Trial registration:**

ISRCTN ISRCTN14957538. Registered in October 2022.

**Supplementary Information:**

The online version contains supplementary material available at 10.1186/s13063-024-08189-4.

## Administrative information

Note: the numbers in curly brackets in this protocol refer to SPIRIT checklist item numbers. The order of the items has been modified to group similar items (see http://www.equator-network.org/reporting-guidelines/spirit-2013-statement-defining-standard-protocol-items-for-clinical-trials/).

**Table:** 

Title {1}	Defatting of donor transplant livers during normothermic perfusion – a randomised clinical trial: Study protocol for the DeFat study
Trial registration {2a and 2b}.	The trial was registered with the ISRCTN registry in October 2022 with the identifier ISRCTN14957538
Protocol version {3}	SPIRIT guidance: Date and version identifier. v3.3; 01/03/2024
Funding {4}	NIHR Efficacy and Mechanism Evaluation Awards (NIHR131163)
Author details {5a}	SPIRIT guidance: Affiliations of protocol contributors. Mr Syed Hussain Abbas Clinical Research Fellow Nuffield Department of Surgical Sciences University of Oxford The Churchill Hospital Oxford, OX3 7LJ E-mail: hussain.abbas@nds.ox.ac.uk Mr Carlo Ceresa Royal Free London NHS Foundation Trust The Royal Free Hospital Pond St, Hampstead London NW3 2QG E-mail: carlo.ceresa1@nhs.net Professor Leanne Hodson Oxford Centre for Diabetes, Endocrinology and Metabolism University of Oxford The Churchill Hospital Oxford, OX3 7LJ E-mail: leanne.hodson@ocdem.ox.ac.uk Mr David Nasralla Royal Free London NHS Foundation Trust The Royal Free Hospital Pond St, Hampstead London NW3 2QG E-mail: david.nasralla@nhs.net Professor Christopher Watson University of Cambridge Department of Surgery Box 202 Addenbrooke's Hospital Hills Road Cambridge, CB2 2QQ E-mail: cjew2@cam.ac.uk Mr Hynek Mergental Queen Elizabeth Hospital University Hospitals Birmingham NHS Foundation Trust Mindelsohn Way, Birmingham B15 2TH TransMedics Inc 200 Minuteman Road, Andover United States MA 01810 E-mail: hmergental@transmedics.com Professor Constantin Coussios Institute of Biomedical Engineering Old Road Campus Research Building University of Oxford Oxford, OX3 7DQ E-mail: constantin.coussios@eng.ox.ac.uk Dr Fotini Kaloyirou NHSBT CTU Long Road Cambridge CB2 0PT Email: fotini.kaloyirou@nhsbt.nhs.uk Dr Kerrie Brusby NHSBT CTU Long Road Cambridge CB2 0PT Email: kerrie.brusby@nhsbt.nhs.uk Dr Ana Mora Victor Phillip Dahdaleh Heart and Lung Research Institute University of Cambridge Cambridge Biomedical Campus Hills Road Cambridge CB2 0BB Email: ana.mora@nhs.net Dr Helen Thomas NHS Blood and Transplant Clinical Trials Unit Fox Den Road Stoke Gifford Bristol BS34 8RR Email: helen.thomas@nhsbt.nhs.uk Dr Daphne Kounali Oxford Clinical Trials Research Unit (OCTRU) Centre for Statistics in Medicine (CSM) Nuffield Department of Orthopaedics, Rheumatology and Musculoskeletal Sciences (NDORMS) Medical Sciences Division The Botnar Research Centre University of Oxford Windmill Road Oxford OX3 7LD Email: daphne.kounali@ndorms.ox.ac.uk Katie Keen NHSBT CTU Long Road Cambridge CB2 0PT Email: katie.keen@nhsbt.nhs.uk Professor Joerg-Matthias Pollok Royal Free London NHS Foundation Trust The Royal Free Hospital Pond St, Hampstead London NW3 2QG Email: Joerg-matthias.pollok@nhs.net Mr Rohit Gaurav Addenbrooke's Hospital Cambridge University Hospitals NHS Foundation Trust Hills Road Cambridge, CB2 0QQ Email: Rohit.Gaurav@nhs.net Mr Satheesh Iype Royal Free London NHS Foundation Trust The Royal Free Hospital Pond St, Hampstead London NW3 2QG Email: satheesh.iype1@nhs.net Mr Wayel Jassem Kings College Hospital King's College Hospital NHS Foundation Trust Denmark Hill London, SE5 9RS Email: wayel.jassem@kcl.ac.uk Professor Thamara Perera Queen Elizabeth Hospital University Hospitals Birmingham NHS Foundation Trust Mindelsohn Way Edgbaston Birmingham, B15 2GW Email: Thamara.Perera@uhb.nhs.uk Mr Abdul Rahman Hakeem St James's University Hospital Leeds Teaching Hospitals NHS Trust Beckett Street Leeds, LS9 7TF Email: abdul.hakeem1@nhs.net Mr Simon Knight Senior Clinical Research Fellow Honorary Consultant Transplant Surgeon Nuffield Department of Surgical Sciences University of Oxford The Churchill Hospital Oxford, OX3 7LJ E-mail: simon.knight@nds.ox.ac.uk Professor Peter J. Friend Professor of Transplantation Director of Oxford Transplant Centre Nuffield Department of Surgical Sciences University of Oxford The Churchill Hospital Oxford, OX3 7LJ E-mail: peter.friend@nds.ox.ac.uk
Name and contact information for the trial sponsor {5b}	University of Oxford University Offices Oxford OX1 2JD England United Kingdom rgea.sponsor@admin.ox.ac.uk
Role of sponsor {5c}	SPIRIT guidance: Role of study sponsor and funders, if any, in study design; collection, management, analysis, and interpretation of data; writing of the report; and the decision to submit the report for publication, including whether they will have ultimate authority over any of these activities. N/A This study is managed by the NHS Blood and Transplant (NHSBT) Clinical Trials Unit. Funding was granted by NIHR EME following a two-stage application review. The sponsor reviewed and approved the study protocol and trial related documents prior to submission for REC approval. However, the sponsor and funder do not have a direct role in study design; data collection, management, analysis, and interpretation of data; writing of the report; and decision to submit the report for publication.

## Introduction

### Background and rationale {6a}

#### Hepatic steatosis and liver transplantation

Liver disease kills almost 11,000 people annually, a 400% increase since 1970 [1]. For many patients, liver transplantation is a highly successful treatment, but its benefit is critically limited by a shortage of donor organs. As a result, up to 20% of patients die on the waiting list before receiving a transplant [2].

In order to increase the donor pool, more sub-optimal ‘marginal’ donor livers are being transplanted [3]. These include livers with substantial intra-cellular fat accumulation (steatosis). Steatosis results from altered metabolism of fatty acids within hepatocytes and is characterised by cytoplasmic accumulation of triglyceride (TG) in the form of lipid droplets (LDs) [4]. Large cytoplasmic LDs cause peripheral displacement of the cell nucleus resulting in macrovesicular steatosis (MaS). Livers with MaS are much more susceptible to post-transplant ischaemia-reperfusion injury (IRI), with the primary initiating event occurring during storage at ice temperature (static cold storage; SCS). The consequent organ injury, attributed to impaired microcirculation, reduced mitochondrial function, and excessive inflammatory response, is associated with poor post-transplant outcomes [5].

There is evidence that moderate to severe steatosis (more than 30% MaS) is associated with primary non-function and a 71% increase in risk of graft loss, and such high-risk organs are frequently declined for transplantation [6]. Around 1000 steatotic livers are retrieved but discarded for this reason each year in the USA [7]; in the UK, 39% of liver discards are primarily due to steatosis [8].

The prevalence of hepatic steatosis, which is commonly associated with obesity, is increasing and currently affects 33% of the UK population [9, 10]. Increasing obesity in the population is reflected in the donor pool; 29% of deceased donors in the UK have a body mass index (BMI) >30 kg/m^2^ [11]. Steatosis in donor livers is increasing and methods to render these organs suitable for transplantation are urgently needed.

NHS Blood and Transplant (NHSBT) has been highly successful in increasing organ donation rates in order to meet waiting list demands, but the quality and utilisation of donated organs is now a key concern. We hypothesise that through the reduction of IHTG content, steatotic livers can be optimised for safe transplantation. This will benefit liver transplant patients by reducing waiting list mortality and post-operative complications. It will reduce the economic burden of chronic liver disease on the NHS and society and maximise the benefit of every donor’s generous gift.

#### Normothermic machine perfusion (NMP) in liver transplantation

Conventional storage of donor organs between retrieval from the donor and implantation in the recipient involves cooling on ice to 4°C (to reduce metabolic activity) and the use of specialist solutions (to reduce cellular swelling). Recently, the benefits of normothermic perfusion have been shown [12–14]; normal physiological functions are maintained during preservation by using a blood-based perfusate at body temperature (37°C) and providing oxygen and nutrients.

This has several benefits: (i) recovery from acute injury (hypoxia) sustained prior to or during retrieval [12]; (ii) objective assessment of organ function prior to transplantation: a number of studies have shown that this enables identification of organs in the ‘high-risk’ category that can safely be transplanted [14–17]; and (iii) extended preservation times (up to 24 h) [14]. Crucially, it also provides the opportunity for therapeutic intervention to a functioning organ before it is transplanted.

Attempts to improve post-transplant outcomes in steatotic livers have included treatments to attenuate the IRI to which these grafts are particularly susceptible. However, in experimental models, levels of injury remained higher in treated steatotic than in lean livers [18–20]. Rather than identifying methods to reduce IRI, targeting the primary cause, accumulation of intra-hepatocellular triglyceride (IHTG), may yield improved transplantation outcomes. By eliminating the root of the problem, the associated complications may be avoided. Several groups have explored this approach, particularly using NMP as a method to enhance the quality of steatotic grafts by actively removing IHTG during preservation.

Our collaborators in Birmingham recently published a systematic review of ex-situ machine preservation of steatotic donor livers, covering both non-pharmacological and pharmacological strategies [21] in their literature search and included studies up to March 2018. Fifteen studies were identified, covering all aspects of machine perfusion (including hypothermic machine perfusion, HMP) relevant to hepatic steatosis and defatting strategies in both animal studies and studies involving discarded human livers.

Out of the 15 original articles, only 4 were relevant to defatting steatotic livers during NMP [22–25]. We undertook a further systematic literature search to identify further experimental and clinical studies relating to defatting interventions during NMP of the liver published since this review and identified two more recent studies [26, 27] (see Table 1).

**Table 1:** Summary of defatting interventions and effect on MaS

Ref.	Defatting interventions	Model	Total ex-situ perfusion time (h)	Percentage (%) reduction in macrovesicular steatosis (MaS)
Nagrath et al. 2009 [22]	GW501516, GW7647, forskolin, hypericin, visfatin and scorparone	Zucker rats (*N* = 12)	3	50
Jamieson et al. 2011 [23]	NMP alone	Porcine (*N* = 8)	48	13
Raigani et al. 2019 [26]	GW501516, GW7647, forskolin, hypericin, visfatin, scorparone and L-carnitine	Zucker rats (*N* = 18)	6	33
Banan et al. 2016 [25]	L-carnitine and exendin-4	Discarded human livers (*N* = 2)	8	10
Liu et al. 2018 [24]	NMP alone	Discarded human livers (*N* = 10)	24	-
Boteon et al. 2019 [27]	GW501516, GW7647, forskolin, hypericin, visfatin, scorparone and L-carnitine	Discarded human livers (*N* = 10)	6	40
			12	50

#### NMP, hepatic steatosis and pre-clinical animal studies

Steatotic livers constitute the largest individual cohort of organs which might be salvaged through active intervention during NMP [22, 23, 25, 26, 27, 28, 29]. Pre-clinical models demonstrate that ex-situ liver function can be enhanced and IHTG content reduced using NMP [22, 23].

Jamieson et al. [23] investigated the effect of NMP alone on steatotic porcine livers during 48-h perfusions. Steatotic porcine livers maintained perfusate base excess, factor V and bile production during NMP and demonstrated comparable haemodynamics and markers of liver injury to lean controls. MaS was reduced from 28 to 15% with reduction in lipid droplet size by the end of preservation [23]. This study demonstrated mobilisation of fat from the liver into the perfusate. Indeed, one limitation of the study was the recirculation of secreted TGs in the circuit, thereby making the perfusate extremely lipaemic. It was thought that this might be a factor that limited the amount of fat that could be extracted by perfusion alone.

Nagrath et al. [22] used an experimental oxygenated normothermic model to investigate the effect of a ‘defatting cocktail’ on steatotic livers from Zucker rats over 3-h perfusions. The ‘defatting cocktail’ combined six pharmacological agents (see Table 2). IHTG content was reduced by 65% with increased hepatic lipid metabolism. Notably, a 30% reduction in IHTG content was seen in the control group with no defatting agents.

**Table 2:** Defatting agents used in Nagrath et al [22]

Defatting agent	Function
PPARδ ligand GW501516	Increase fatty acid β-oxidation
Peroxisome proliferator-activated receptor (PPAR) α ligand GW7647	Increase mitochondrial fatty acid oxidation
Cyclic adenosine monophosphate (cAMP) activator forskolin	A glucagon mimetic cAMP activator, increases lipolysis and fatty acid oxidation
Pregnane X receptor ligand hypericin	Increase β-oxidation (very long chain fatty acids)
Visfatin	An insulin-memetic adipokine, role not fully understood
Scorparone	An androstane receptor ligand, upregulates PPAR

Raigani et al. [26] demonstrated similar results in a Zucker rat model using the addition of L-carnitine (to increase fatty acid β-oxidation) to the ‘defatting cocktail’ described by Nagrath et al. [22]. MaS was reduced from 41.5 to 8.5% during defatting perfusion over 6 h. There was an increase in perfusate ketone content (a marker of fatty acid β-oxidation), bile bicarbonate content and lactate clearance in treated livers.

These pre-clinical animal studies show the potential of NMP as a platform to deliver targeted intervention(s) to treat donor hepatic steatosis, with evidence that both IHTG and MaS can be reduced during ex-situ NMP. However, these studies involved artificially induced steatosis and it is not clear how well this replicates the clinical situation and whether a clinically-relevant effect is achievable in steatotic human livers.

#### NMP, hepatic steatosis and discarded human livers

Liu et al*.* [24] perfused ten discarded livers with variable degrees of baseline steatosis for 24 h and demonstrated a significant increase in perfusate TG levels over the duration of the perfusion, suggesting mobilisation of IHTG. However, no histological reduction in IHTG content was observed. Banan et al. reported results from two human livers which were preserved normothermically with two defatting agents (L-carnitine and exendin-4); one of these showed a 10% reduction in the degree of MaS after 8 h NMP [25].

**Fig. 1:**
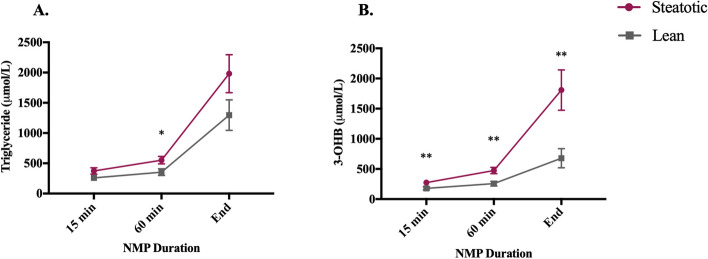
**A–B** Comparison of circulating TG (**A**) and 3-OHB (**B**) in the perfusate during NMP between steatotic (*n* = 18) and lean (*n* = 15) livers. Data presented as mean ± SD. *p < 0.05, **p < 0.01

**Fig. 2:**
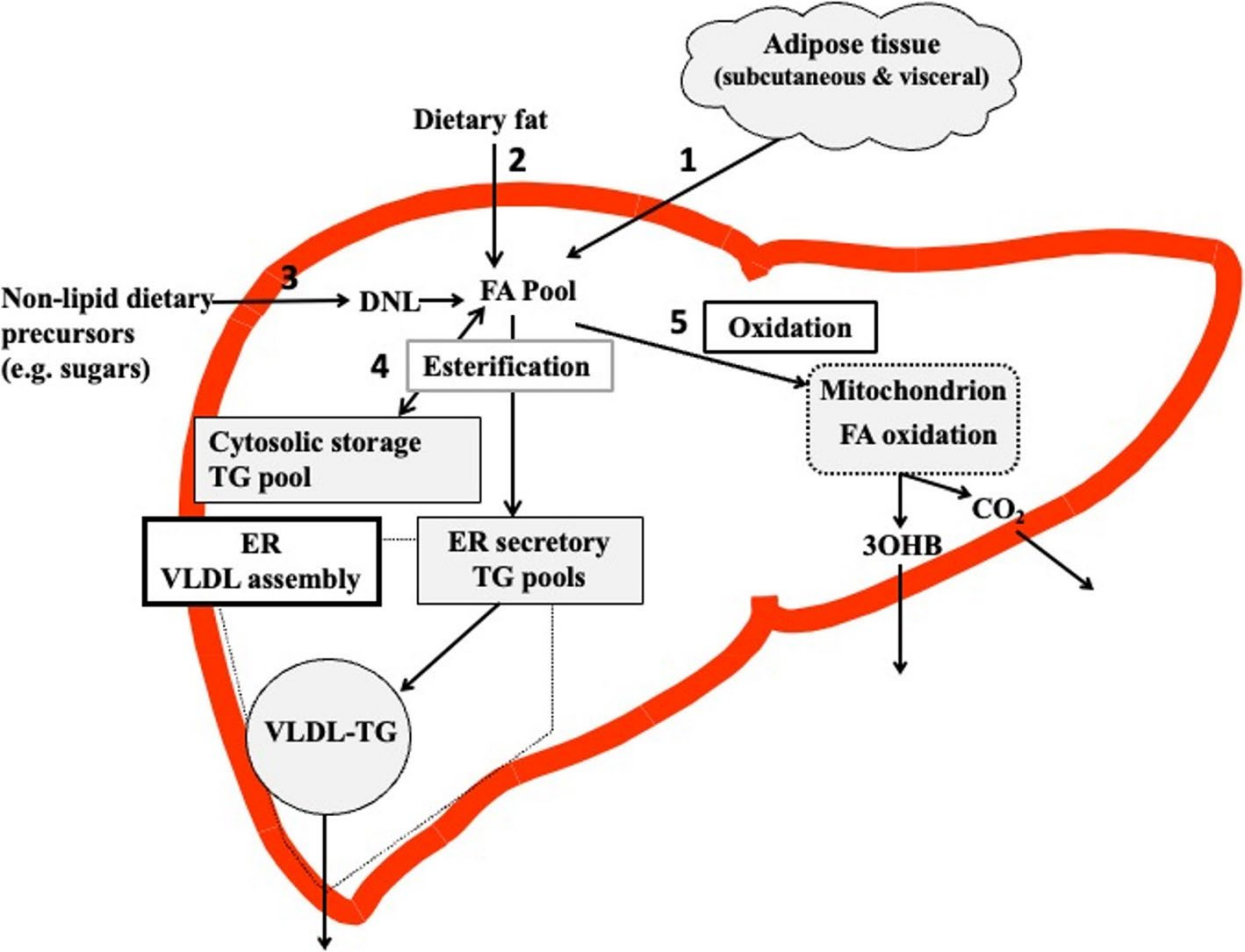
Overview of hepatic fatty acid (FA) input, synthesis and disposal in the postprandial state. FA input to the liver derives from 1) the lipolysis of adipose (subcutaneous and visceral) tissue triglyceride (TG), and 2) dietary fat, which enter the liver as either chylomicron remnants or chylomicron-derived spillover FAs. 3) FA synthesis occurs within the liver, via de novo lipogenesis (DNL) which involves the synthesis of FA from acetyl-CoA derived from non-lipid precursors, such as glucose. FAs enter a common pool and are broadly partitioning between two pathways for disposal: 4) the esterification pathway, where predominantly TG is produced which can then be either stored in the cytosol (as a lipid droplet) or can lipidate very-low density lipoprotein (VLDL) in the endoplasmic reticulum (ER) to form VLDL-TG and then secreted into the systemic circulation, or 5) oxidation either via the tricarboxylic acid (TCA) cycle to form CO2, or the ketogenic pathway where β-hydroxybutyrate (3OHB) is produced and enters the systemic circulation

When our group explored the effect of NMP (alone) on transplanted steatotic human livers (as part of a larger trial), we observed clear differences in TG metabolism during preservation compared to lean livers. As with previous groups [24, 25, 27], we observed significant increases in perfusate TG and 3-hydroxybutyrate (3-OHB) (a marker of hepatic fatty acid oxidation) during perfusion (Fig. 1) [29]. This suggests that a steatotic liver upregulates pathways to dispose of intrahepatic fatty acids, including mobilising and secreting more very low-density lipoprotein (VLDL)-TG and increasing ketone body production (Fig. 2). Despite these changes in TG metabolism, the amount of IHTG did not change when assessed histologically. Furthermore, although steatotic NMP livers demonstrated significantly superior post-transplant biochemical function compared to steatotic cold-stored livers (implying less preservation injury), there was still evidence of greater injury than in lean counterparts, with a significantly higher post-operative peak serum aspartate aminotransferase (AST) level (*p* = 0.02) [29].

Further evidence supporting the need for targeted intervention for steatotic livers beyond NMP alone comes from our collaborators in Birmingham. Previously declined livers were perfused and those meeting pre-defined functional criteria were transplanted: 22 of 31 perfused organs were transplanted, all with immediate function [17]. Notably, of the livers that did not meet ‘viability criteria’ and were therefore not transplanted, 71% had histological evidence of moderate to severe steatosis. These data suggest that steatotic livers require more active intervention beyond simply replacing static cold storage with NMP.

Using the same ‘defatting cocktail’ as that of Nagrath et al. [22] with the addition of L-carnitine, Boteon et al. [27] treated organs retrieved for clinical transplantation but discarded due to steatosis. Using groups of five livers (only four in each group had evidence of MaS), pharmacological intervention was associated with improved metabolic function, reduced vascular resistance, lower levels of liver injury and increased bile production. There was evidence of reduction in markers of oxidative injury, immune cell activation, release of inflammatory cytokines and tissue TG. There was 40% reduction of MaS at 6 h of perfusion. All 5 livers that received defatting therapies achieved viability criteria for transplantation compared to 2/5 in the control group (*P* = 0.04). However, not all treated livers that met the viability criteria achieved the clinical threshold of MaS of <30% [27]. This calls into question the correlation between histology and function. It is possible that activation of cytoprotective and vaso-protective pathways are the key elements that render such organs suitable for transplantation [28]. NMP and defatting may have synergistic effects in achieving viability criteria for transplantation.

Although Boteon et al*.* demonstrated favourable outcomes, this was in a pre-clinical study and did not include livers that were transplanted. Careful evaluation of the safety profile of this proposed ‘defatting cocktail’ is required prior to clinical use. Currently, many of the agents included in the ‘defatting cocktail’ lack important safety data (although there is some cytotoxicity tested reported in vitro) [30]. Hypericin is a component of St John’s Wort that is involved in upregulation of the cytochrome P450 3A4 enzyme [31]. This enzyme is involved in the metabolism of medications including cyclosporine and tacrolimus. In addition, the peroxisome proliferator-activated receptor agonists GW501516 and GW7647 have not been tested in human trials and concern has been raised regarding carcinogenesis in preliminary animal studies [32].

Recent work from our own group (currently unpublished) has been directed to treating steatotic livers with the intention that these should be made to function as well as lean counterparts, by means of active intervention to remove fat during preservation [33]. Whilst designing this research, we considered the requirements of subsequent clinical translation and therefore avoided use of unlicensed chemical compounds which would require extensive testing and optimisation prior to use in a clinical trial. We also considered the conclusions of our earlier porcine perfusions and the potential benefit of removing mobilised fat from the perfusate [23, 33].

Our preliminary results in organs retrieved for clinical transplantation but discarded due to steatosis have demonstrated the potential of a novel defatting strategy [33]. Using the commercially available OrganOx *metra*^*®*^ device, 18 livers were perfused: 6 using a standard NMP protocol (Group 1); 6 using a circuit including a lipoprotein apheresis filter to remove circulating lipids (Group 2), and 6 using the lipoprotein apheresis filter and pharmacological interventions (Group 3). All livers were perfused over 48 h.

**Fig. 3:**
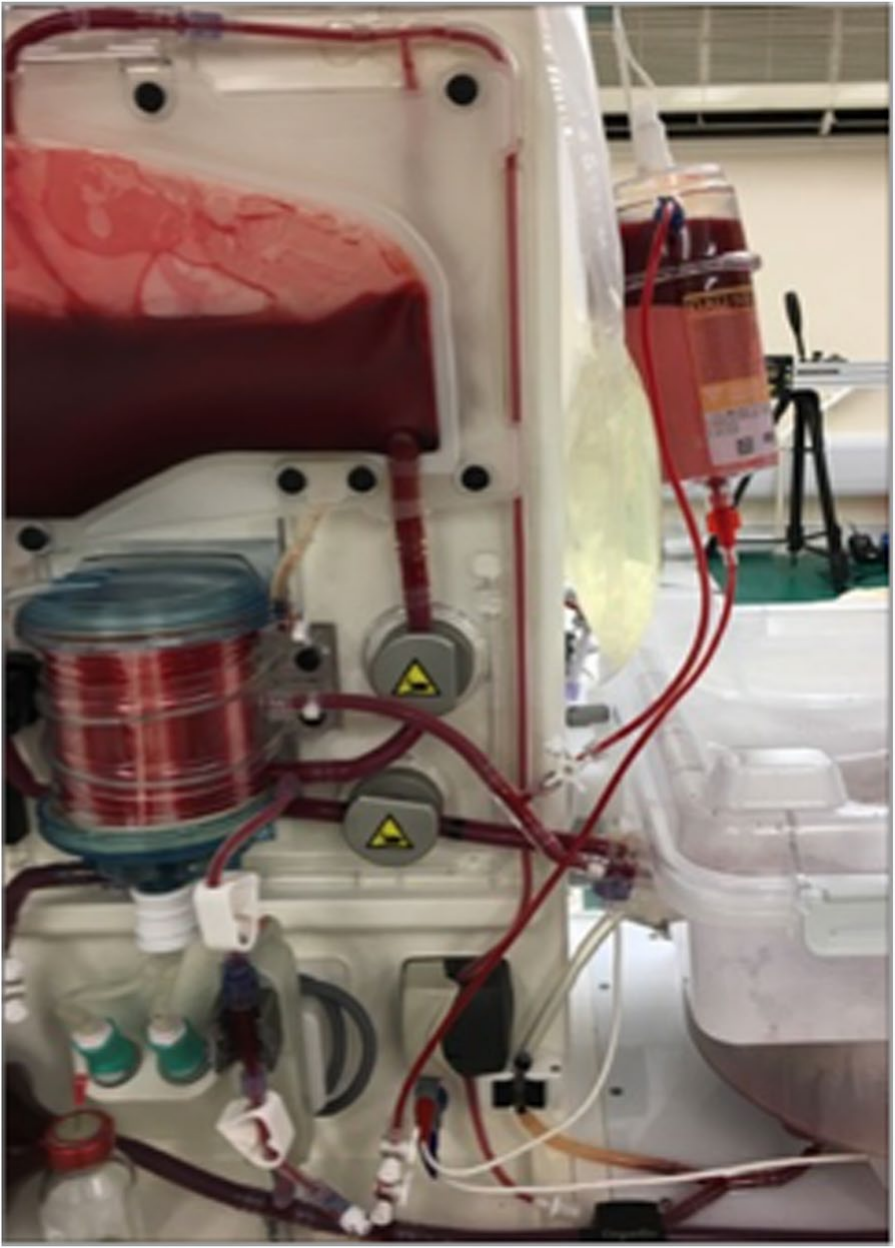
Lipoprotein apheresis filter in NMP circuit

The first intervention was aimed at reducing the amount of VLDL-TG circulating in the perfusate; these are thought to be pro-inflammatory and might contribute to on-going IHTG accumulation (these can be recycled through the liver). To remove VLDL, a lipoprotein apheresis (DALI^®^ 500) filter (Fresenius Medical Care (UK) Ltd, Huthwaite, UK) was incorporated into the circuit (Fig. 3). In clinical practice, this haemofiltration system is used for patients with severe hyperlipidaemia, refractory to medical therapy [34]. The filter consists of a matrix of polyacrylate beads, effective for the adsorption of cholesterol, lipoprotein (a) and TGs [34]. Following this, we further modified the perfusate to include the following interventions:

1. *L-carnitine*: is comprised of amino acids including lysine and methionine. It is naturally present in meat, fish dairy products and plants [35]. Humans can synthesise carnitine; therefore, its availability is not limited to dietary intake [36]. L-carnitine can increase the rate of fatty acid transport to mitochondria and is important in β-oxidation of fatty acids from the mitochondrial membrane [36]. For this reason, it has been proposed as a weight loss supplement. It is licensed for use in primary carnitine deficiency due to inborn errors of metabolism and prevention of L-carnitine deficiency in patients with kidney disease undergoing haemodialysis.

The perfusate was supplemented with 1 g of L-carnitine hydrochloride. This dose was based on in vivo human studies investigating the effect of L-carnitine in the treatment of hyper-lipoproteinemia, chronic myocardial ischaemia and deficiency in paediatric patients on peritoneal dialysis. The intravenous pharmacokinetics were determined from an in vivo study involving healthy subjects on a low-carnitine diet [37–40]. L-carnitine has a half-life of around 15 h [41]; therefore, a further 1 g was administered at 24 h of perfusion.

Efficacy: L-carnitine has been investigated in cardiovascular disease and type 2 diabetes studies, in which plasma lipid levels and weight loss were secondary outcomes. In a trial of 258 patients with uncontrolled type 2 diabetes, 2 g/day of L-carnitine with orlistat (360 mg/day) for 1 year significantly increased weight loss compared to orlistat alone [42]. A recent metanalysis of 911 patients showed an average 1.33 kg excess weight loss compared to placebo. The doses administered ranged from 1.8 to 4 g/day [36].

Safety: L-carnitine supplements are generally well tolerated at doses of up to 4 g/day. Some side effects reported during in vivo human studies include nausea, vomiting and increased frequency of bowel movement. Rarer side effects reported include muscle weakness in patients with uraemia and seizures (in patients with underlying seizure disorders) [43, 44].

2. *Forskolin*: is a dietary supplement originating from the roots of *Coleus forskohlii*, a plant prevalent in India and Thailand. It has reported to facilitate weight loss through lipolysis and appetite suppression [45, 46]. It is a glucagon mimetic cAMP activator which results in increased lipolysis and fatty acid oxidation [47].

The perfusate was supplemented with 1 mg NKH477 hydrochloride (a water-soluble version of forskolin), a dose based on data from previous studies in patients with cardiomyopathy [48] and schizophrenia [49] at doses of 0.1–0.5 mg/kg.

Efficacy: Forskolin has demonstrated suppression of appetite in pre-clinical animal studies. A randomised double-blind trial showed significant (4%) reduction in body fat in 30 overweight men compared to placebo [46].

Safety: During in vivo human studies, forskolin has been reported to increase frequency of bowel movement. Doses of 500 mg/day have not been associated with any serious or adverse events [50].

3. *Insulin reduction*: De novo lipogenesis (DNL) is the process through which the liver synthesises fatty acid, namely the 16-carbon saturated fatty acid palmitate, from non-lipid precursors. This is stimulated by insulin [51]. Enhanced DNL may have significant effects on cellular metabolism as the primary fatty acid product is saturated (palmitoyl-CoA) [52, 53] which may interfere with cellular function [54]. In the absence of peripheral fat stores or dietary fat in this model, the only source of fatty acid production in the liver is via the DNL pathway. In order to lower DNL, we reduced the amount of insulin delivered during the perfusion by 50%. The perfusate was infused with 100 units of Actrapid, dissolved in 30 mL 0.9% NaCl, at 1 mL/h.
4. *Glucose reduction*: Glucose acts as a non-lipid precursor for DNL [55]. In order to reduce the liver’s ability to de novo synthesise fatty acids, the glucose threshold to commence infusion of parenteral nutrition (TPN) infusion was reduced from 10 mmol/L to 5 mmol/L to reduce perfusate glucose concentration.

**Fig. 4:**
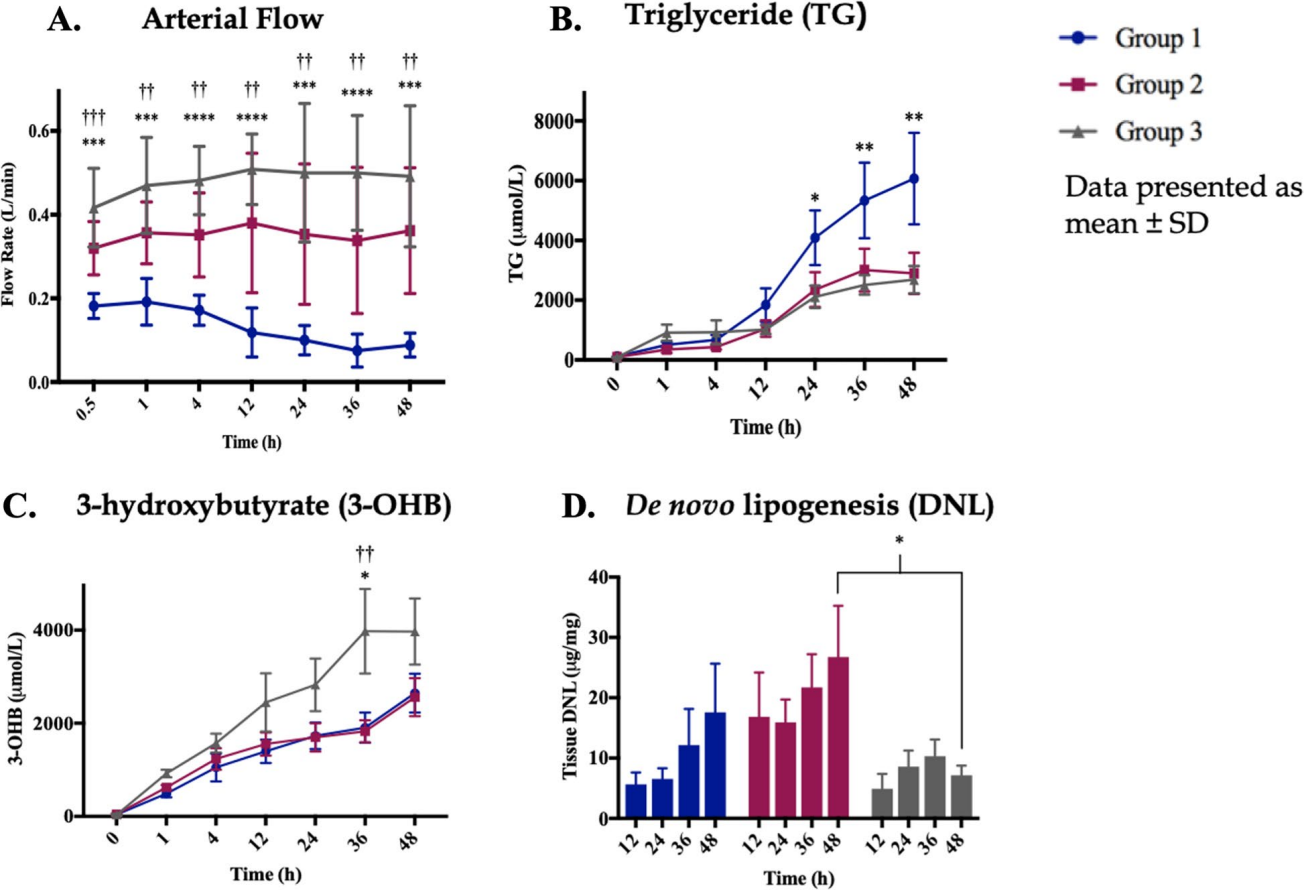
**A–D** Group 1 NMP alone, Group 2 filter and Group 3 filter plus defatting agents

Over the 48-h perfusion, a significantly increased arterial flow was seen in both intervention groups (Groups 2 and 3) (Fig. 4A). The lipoprotein apheresis filter (Groups 2 and 3) significantly reduced circulating TG concentrations (Fig. 4B). 3-OHB measurements showed a significant increase in fatty acid β-oxidation in Group 3, where L-carnitine and forskolin had been added (Fig. 4C). Significant reduction in intrahepatic DNL (as measured in liver tissue using stable-isotope methodology) was seen in Group 3 (Fig. 4D). Other functional benefits observed in both Groups 2 and 3 were increased hepatic glycogen production, less rise in perfusate transaminase and a reduction in haemolysis (which we previously identified as a marker of ex-situ liver function [14]).

The combination of lipoprotein apheresis filtration and defatting interventions significantly reduced the amount of fat within the liver by 45% at 48 h. However, the functional improvements were seen much earlier, by 6 h. From this experimental study, we concluded that a combination of lipoprotein apheresis filtration and perfusate modification reduces hepatic steatosis and improves ex-situ liver function.

Overall, the pre-clinical studies of ex-situ defatting in discarded human livers, from both Oxford and Birmingham, have demonstrated a ‘proof of concept’ [27, 33]: if this translates into clinical practice, it will significantly increase the number of safely transplantable organs by increasing utilisation of ‘marginal’ organs. The interventional agents proposed are safe, well-tolerated and available for clinical use (in contrast to previous studies) [22, 27]. We do not anticipate any systemic side effects following transplantation; first, because the doses are much lower than those used in human studies and, second, because these agents will be administered ex-situ to livers that are then thoroughly flushed prior to transplant (as per standard practice following NMP) removing the agents from the liver prior to transplantation.

In our discarded liver study, structural and functional differences were evident after 6 h of perfusion. These were associated with improved perfusion and biochemical metrics that would have rendered these organs transplantable on current functional criteria. This suggests that 6 h of perfusion should be the minimum required prior to considering implantation of the organ into the recipient (with a maximum of 24 h as per OrganOx *metra*^*®*^ instructions for use).

In the proposed trial, we intend to enrol livers that have been retrieved for the purpose of transplantation and that have been identified as high-risk of steatosis. We know that such livers are likely to be discarded after retrieval either because of appearance, histology or unfavourable perfusion metrics on NMP. We will test the targeted defatting protocol described above, using objective measures of function to assess outcomes.

## Objectives {7}

The experimental data outlined above suggest that the combination of NMP and defatting may be effective in reducing the fat content of livers and improving perfusion parameters to meet functional criteria for transplantation. In this first clinical study, the primary objective is to confirm the safety and assess efficacy of the NMP-defatting protocol in steatotic donor livers intended for transplant. The secondary objective is to test the feasibility of the (i) inclusion criteria (false positives and negatives); (ii) delivery of intervention and (iii) the study endpoints. These objectives will provide information of likely effect sizes in order to design a subsequent phase III study in the future.

The mechanistic studies will be analysed subsequent to the main clinical outcomes. These will be carried out for two broad reasons (i) to identify more sensitive and specific markers of transplantability and (ii) to understand the process of defatting that leads to a steatotic organ being reconditioned. The proposed mechanistic studies and associated outcome measures are described in the outcome section of this protocol.

## Trial design {8}

This is a prospective, blinded randomised study, which will test the effect of normothermic defatting of steatotic donor livers. Donor organs meeting enrolment criteria will be randomised, using a 1:1 allocation ratio, using permuted blocks of varying undisclosed size and will be stratified by donor organ type (DBD/DCD). Livers will be perfused using the OrganOx *metra*^*®*^ NMP device and assigned to either NMP alone (*n* = 30) or NMP with defatting interventions (*n* = 30). An interim safety review will be undertaken after perfusion of the first ten livers.

All recruiting centres have extensive experience in the clinical use of NMP and are current users of the OrganOx *metra*^*®*^ device. The OrganOx *metra*^*®*^ is a CE marked normothermic preservation device for use in human liver transplantation. It perfuses the donor liver with the blood, oxygen and nutrients, as well as a number of medications, at normal body temperature to replicate physiological conditions and preserve the organ for up to 24 h. The device provides information as to the haemodynamic, synthetic and metabolic function of the liver during perfusion, which may assist the clinician in assessing the organ’s suitability for transplantation. The device is available at all recruiting liver transplant centres.

Perfusions will be supervised by a member of the central trial team. Randomisation (through the web-based service, www.sealedenvelope.com) will be carried out by the trial co-ordinator (clinical research fellow) after inspection of the organ with the transplanting surgeon. Following randomisation, setting up the NMP device will follow standard practice, with addition of the apheresis filter and pharmacological protocol (see below). The presence or absence of the lipoprotein apheresis filter will be blinded through use of a ‘dummy’ filter covered by a drape. This will prevent the local transplant team (and therefore the patient) from knowing the study allocation. An interim safety review will be undertaken after perfusion of the first ten livers.

Study visits will align with routine outpatient clinics to avoid extra hospital visits where possible. These will be at post-operative days 1–7, day 30 and months 3 and 6. At each study visit, details of adverse events, biochemical liver function tests and graft and patient survival will be documented.

The collaboration with the NHSBT Clinical Trials Unit (CTU) will facilitate longer-term (12 month) follow-up of basic parameters (where data is available) beyond the end of the trial and we will request consent to do so. This data will be collected from the UK Transplant Registry (UKTR) held by NHSBT.

Data will be collected into a secure central online electronic database (MACRO) using electronic case report forms. The study will close after the final patient has completed 6 months of follow-up. Longer-term (12 month) follow-up data (beyond the end of the trial) will be collected from the UKTR.

## Methods: participants, interventions and outcomes

### Study setting {9}

Recruitment will take place at five UK liver transplant centres (Addenbrooke’s Hospital, Cambridge; King’s College Hospital, London; Queen Elizabeth Hospital, Birmingham; Royal Free Hospital, London; St James’s Hospital, Leeds). All of these centres have extensive experience in the clinical use of NMP and are current users of the OrganOx *metra*^*®*^ device.

### Eligibility criteria {10}

#### Trial participants

The randomised entity in this study is a donor liver, rather than a transplant recipient. Donor livers accepted by each participating transplant centre will be screened for a high likelihood of fatty liver disease at each point of the donor pathway based on (i) waist circumference (>88 females and >102 cm males) or BMI >30 kg/m^2^ or both at point of acceptance [56] and (ii) evidence of macroscopic moderate-severe steatosis identified by the retrieval surgeon (or biopsy result) at point of retrieval.

In addition, any liver offer fast tracked due to moderate–severe steatosis (based on appearance or biopsy result) will also be considered for enrolment (regardless of WC and/or BMI).

The final entry criterion will occur at the point of inspection upon arrival at the transplant hospital: a surgeon from the implanting team will assess the liver to confirm its suitability for inclusion into the trial (based on macroscopic characteristics: colour, texture, rounded edges, size, weight) [57]. The objective of this second entry criterion is to reduce the number of false positive (non-fatty) livers enrolled in the trial. Where available, the results of clinical biopsies demonstrating moderate-severe steatosis (typically >30%) will also be taken into account to assess suitability for randomisation.

Outcomes of livers transplanted during the study will be assessed. Liver transplant recipients will be those on the waiting list in participating centres to whom the liver is offered, and recipients will be consented for use of their data. This study does not alter the normal UK offering process in any way.

#### Inclusion criteria

Donor livers:

- Donors aged 18 years or over
- Offered through the national offering scheme and accepted by participating liver transplant centre
- Moderate–severe steatosis: macroscopic characteristics based on colour, texture, rounded edges, size and weight at point of inspection at the transplant hospital to confirm suitability for randomisation. Where available, the results of clinical biopsies demonstrating moderate–severe steatosis (typically >30%) will also be taken into account to assess suitability for randomisation.

Liver transplant recipients:

- Recipients 18 years of age or above
- Elective waiting list at a participating centre
- Willing to consent for inclusion into the study and collection and use of their data

#### Exclusion criteria

Donor livers:

- Donors from outside of the UK
- Donor is HIV, hepatitis B or C positive
- Cold ischaemia time (CIT) expected to exceed > 10 h
- Macroscopic evidence of fibrosis
- Livers undergoing any other form of ex-situ machine preservation
- Participating centre cannot offer NMP due to device, logistical or staffing reasons

Liver transplant recipients:

- Receipt of a liver that has not undergone randomisation
- Receipt of super urgent transplant for acute liver failure
- Receipt of a split liver transplant
- Receipt of a multi-organ transplant
- Transplanted outside of the participating centres
- Contraindication to MRI, e.g. pacemaker

#### Recruitment

All UK liver offers meeting the inclusion criteria will be eligible for consideration. Offers are managed by NHS Blood and Transplant Hub Operations using the electronic offering system (EOS). Following NHSBT standard practice, potential donors are identified by the donor hospital ITU staff and referred to the specialist nurse for organ donation (SNOD). The SNOD will obtain donor family consent for donation, and/or research samples, arrange any necessary investigations and register the donor with Hub Operations as per standard practice.

Liver offering will follow standard NHSBT policy, and offering will not be altered in any way by participation in the study [58].

#### Screening and eligibility assessment

**Fig. 5:**
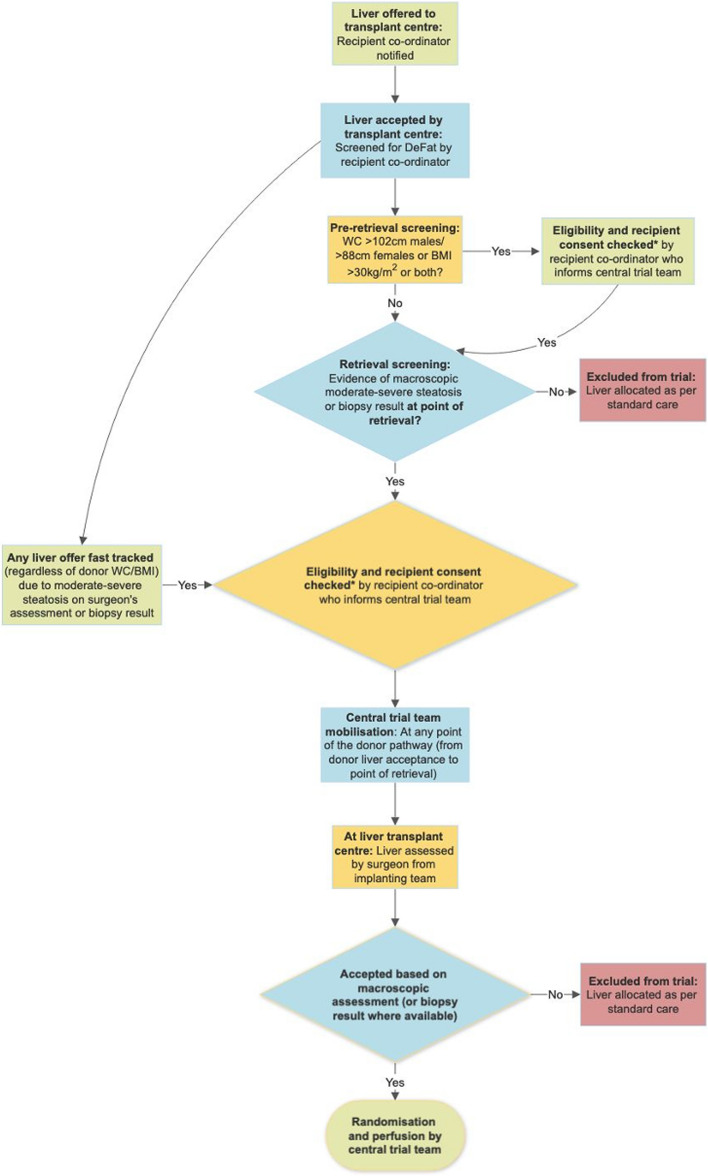
Eligibility screening of donor livers and eligible participants for the DeFat study. *Consent refers to evidence of a signed informed consent form. If a patient on the waiting list has indicated intention to consent and has not returned the signed consent form prior to admission for transplant, they will be asked to sign and confirm consent on admission for transplant. Randomisation and perfusion will only be carried out if the informed consent form has been signed

Screening of donor livers is described above and the anticipated flow of liver offers through the trial is depicted in Fig. 5. Randomisation will be undertaken by the trial co-ordinator (after inspection of the liver by a surgeon from the implanting team). Where available, the results of clinical biopsies demonstrating moderate–severe steatosis (typically >30%) will also be taken into account to assess suitability for randomisation.

At each point of the donor pathway, the recipient co-ordinator from the participating site will communicate relevant information to the trial co-ordinator (clinical research fellow) in order to identify suitable and eligible liver offers. This information will influence the trial co-ordinator's decision to mobilise to the recruiting liver transplant centre. The distance of the donor liver and the central trial (Oxford) team from the recipient liver transplant centre will also influence this decision. The trial co-ordinator will exercise his judgement to avoid prolonged cold ischaemia times (CIT) and any undue delay.

For offers to a named recipient, the recipient co-ordinator will determine whether the recipient has indicated intention to consent or has already consented to the trial. If so, the recipient co-ordinator will contact the central trial team to mobilise to the site. Where an offer is made without a named recipient, the recipient co-ordinator will wait until a recipient is identified in-centre. The central trial team will only be informed if an eligible liver has been allocated to a consented patient (or a patient who has indicated intention to consent on admission for transplant). On arrival to the recipient hospital, the central trial team will only randomise and carry out perfusion providing that there is evidence of signed informed consent. In the absence of consent, the liver will be excluded from the study. Screening logs of all offers will be maintained at each site and will record if donor family consent for research samples has been provided.

Allocation of organs will not be affected in any way by this study. NHSBT matching runs and in-centre allocation of organs will follow usual practice, irrespective of eligibility for the study.

### Who will take informed consent? {26a}

#### Consent procedures

Consent for organ donation and/or research samples from the donor family will be obtained and recorded by the SNOD as per NHSBT standard practice.

All participating liver transplant centres have an OrganOx device available for routine clinical use. Patients in all centres are informed of different methods of preservation at the time of listing and/or explicitly consented for the use of preservation technologies.

Eligible patients on the transplant waiting list in each centre will be approached during a routine outpatient visit or by phone. Written information and a copy of the informed consent form (ICF) will be provided in person, via post or via email. In order to allow sufficient time for considering participation in the study, this initial approach will be followed up by a further phone call or clinic visit at which point a consent discussion will take place and consent will be requested. If followed up by phone call, the patient will be asked to confirm consent by signing the ICF and posting or emailing the signed ICF back to the site team. In cases where email is used, the patient will be requested to scan/photograph and send the signed ICF back by replying to an email sent by the local site team/consenter. Similarly, if the patient is followed up in clinic, they will be asked to sign the ICF in-person during their clinic visit.

If a patient on the waiting list has indicated intention to consent and has not returned the signed consent form prior to admission for transplant, they will be asked to sign and confirm consent on admission for transplant.

In the event of a fast-track liver offer, where the liver may arrive at the site before the patient, there is a chance that randomisation and perfusion of the liver may commence prior to the arrival of the patient. This will only be permitted if a signed ICF has been received from the intended recipient in advance, either by post, email or in-person. In this scenario, the patient will be contacted by telephone prior to randomisation to reaffirm consent.

If the patient chooses to withdraw from the study at any point between consent and randomisation, the liver will not be randomised, it will be excluded from the study and offered as per standard care.

Written and verbal versions of the participant information sheet and informed consent form will be presented to the participants detailing no less than (i) the exact nature of the trial, (ii) what it will involve for the participant, (iii) the implications and constraints of the protocol and (iv) the known side effects and any risks involved in taking part. It will be clearly stated that the participant is free to withdraw from the trial at any time for any reason without prejudice to future care, without affecting their legal rights and with no obligation to give the reason for withdrawal.

The participant will be allowed as much time as possible to consider the information and the opportunity to question the investigator or other independent parties to decide whether they will participate in the trial. Written informed consent will then be obtained by means of dated signatures of the participant and the person who presented and obtained the informed consent. The participant must personally sign and date the informed consent form before any trial-related procedures are performed. The person who obtained the consent must be suitably qualified and experienced and have been authorised to do so by the chief or local principal investigator. A copy of the signed informed consent will be given to the participant. The original signed informed consent form will be retained at the trial site Supplementary materials 2.

If a recipient refuses consent, the liver will be preserved and transplanted according to usual centre practice and will not be randomised into the trial. In addition, patients who are unable to consent for themselves at baseline will not be recruited to this study.

Whether or not a patient consents does not affect organ offering or the chances of receiving a transplant in any way. Once a liver has been offered to the recipient, this offer will be maintained unless the recipient surgeon feels that the liver is not suitable for transplant or the recipient is not medically fit to undergo the procedure. Similarly, if the liver is not deemed eligible for the study by the trial co-ordinator (clinical research fellow), it will be excluded from the trial and offered as per standard care.

Post-operatively, there is a small risk that participants in this study may lose capacity to consent to continued involvement in this study: this incapacity may be transient or (unusually) longer-term. It is routine for patients to be cared for on intensive care or a high dependency unit for a short period following a liver transplant, and they remain sedated following surgery as part of this higher-level care. The risk of a permanent loss in capacity (for example, due to a peri-operative stroke) following a liver transplantation is very low. In the event of a prolonged loss of capacity to consent to continued involvement in the trial, we would provide their designated consultee with information about the study (as the patient information sheet) and seek advice from them as consultee about continuing to collect samples and data from the participant whilst their capacity is impaired.

#### Patients who lack understanding of verbal or written English

Patients and parents/carers with an insufficient understanding of the English language should not be approached to discuss trial participation unless there are adequate arrangements at the site for translation or interpretation of the trial documents. The sponsor is unable to cover the cost of translation due to financial constraints. However, most participating sites will make use of translation services for communication and procedure consent and use of these services is permissible if feasible.

### Additional consent provisions for collection and use of participant data and biological specimens {26b}

A detailed description of the study specific mechanistic studies is provided in section 12. Participants are also consented for the use of their anonymised samples and data in future ethically approved research, i.e. ancillary studies (in the UK or abroad).

## Interventions

All livers in this study will be perfused using the OrganOx *metra*^*®*^. The primary perfusion fluid for the liver comprises packed red blood cells, supplemented by colloid solution (human albumin solution or Gelofusine as per local protocol) to normalise the haematocrit and osmolarity.

Before connection of the liver, the blood-based perfusate is supplemented with:

- Antibiotic and antifungal agents as per current local protocols. Heparin (anticoagulant) to prevent thrombosis in the circuit. In clinical use, a half-life of ~90 min is assumed; on this basis, heparin is also given as a maintenance infusion.
- Sodium bicarbonate (buffer) for adjusting the pH of the perfusate.
- Calcium gluconate/calcium chloride to correct the binding of citrate to calcium

During the perfusion the following are infused at a constant rate:

- Parenteral nutrition solution—a source of amino acids and glucose for liver maintenance.
- Insulin to control the perfusate glucose level
- Heparin to maintain anticoagulation
- A 2% solution of sodium taurocholate in isotonic saline to compensate for loss of bile salts.
- Prostacyclin to optimise microperfusion

All solutions required will be attached to the circuit during set-up and before the liver is attached. All solutions are prepared immediately before the organ is attached to the device and contain sufficient solution for 24 h operation, the intended maximum perfusion time for a liver on the device.

### Explanation for the choice of comparators {6b}

#### NMP (control group)

All livers included in the study will undergo NMP using the OrganOx *metra*^*®*^, a CE-marked device already in use in liver transplant units in both clinical trials and routine clinical practice (providing special arrangement for consent, governance, audit and research) [14, 59].

Livers will be transported on ice to the transplant centre, and those meeting the inclusion criteria will undergo NMP (minimum 6 h, maximum 24 h), in accordance with the manufacturer’s instructions for use and current local protocols. The procedure for preparing the device for use and placing the organ on the device is described in detail in the instructions for use (IFU) document (L300-0437ReV1.0 RoW Version 25/09/2017). All livers will be perfused with three units of donor-type (or O-negative) red blood cells and will be arranged by the recipient surgical team at the recruiting liver transplant centre.

The procedure for removing the liver from the device is also described in the IFU. Implantation and reperfusion of the liver proceed as per the usual practice of the implanting centre.

### Intervention description {11a}

#### NMP with defatting interventions (study group)

The study arm of this trial combines the use of a lipoprotein apheresis filter to the normothermic circuit (for a minimum of 6 h and a maximum of 24 h), with targeted pharmacological strategies during ex-situ perfusion. Therefore, in addition to NMP, livers randomised to the study group will undergo the defatting protocol developed in our previous experimental study [33]. All components of this protocol are licensed for clinical use and are described in detail in the background section of this protocol. Briefly, these include:

- Lipoprotein apheresis filtration: this is licensed for patients with severe hyperlipidaemia refractory to maximal medical therapy [34].
- L-carnitine: this is licensed for use in primary carnitine deficiency due to inborn errors of metabolism and prevention of L-carnitine deficiency in patients with kidney disease undergoing haemodialysis. It has been shown to increase β-oxidation of fatty acids from the mitochondrial membrane. The perfusate will be supplemented with of L-carnitine 1 g/5 mL aqueous solution [37–40].
- Forskolin: This is a glucagon mimetic cAMP activator which results in increased lipolysis of lipid droplets and fatty acid oxidation [47]. The perfusate will be supplemented with 1 mg of NKH477 (water-soluble version of forskolin) in 2 mL of 0.9% sodium chloride (from a stock solution of 5 mg of NKH477 in 10 mL of 0.9% sodium chloride) [48, 49].
- Insulin: this will be infused at a 50% lower concentration than in the OrganOx instructions for use. This reduces the stimulation of de novo lipogenesis (DNL), the only source of fatty acid production in the liver during NMP [51].
- Glucose: the threshold to infuse parenteral nutrition (TPN) will be reduced from 10 mmol/L to 5 mmol/L. As glucose is a non-lipid precursor for DNL, this will reduce the liver’s ability to synthesise fatty acids de novo during perfusion [55].

Normothermic defatting will treat the liver in the ex-situ setting. Following treatment, prior to transplantation, the liver will be flushed with 2 L of preservation solution, as per standard NMP practice. The investigational agents will therefore be effectively removed from the liver prior to implantation.

### Criteria for discontinuing or modifying allocated interventions {11b}

The randomised entity in this study is the donor liver. Randomised livers that are not perfused due to unforeseen reasons will not be replaced. It is anticipated that non-perfusion of a randomised liver will be a very uncommon event

### Strategies to improve adherence to interventions {11c}

Perfusions will be carried out by members of the central trial team who have experience in delivering the intervention from preclinical studies. Delegated staff at each recruiting centre have completed training for imputation of trial related data onto the MACRO™ database. These tasks will be performed under the supervision of the PI at each site. In addition, the DeFat study has utilised the NIHR Associate PI scheme with an identified delegate to support adherence to monitoring of outcome measures. Comprehensive details are also provided in the DeFat study manual summarising trial related procedures.

### Relevant concomitant care permitted or prohibited during the trial {11d}

Following randomisation the liver will be allocated to either the standard NMP protocol or the NMP-defatting protocol. Transplantation of the liver will be based on ex-situ function and at the discretion of the implanting surgeon. Recipient management including the implantation procedure, postoperative care, immunosuppression and other medications, and post-transplant monitoring will follow local protocols.

### Provisions for post-trial care {30}

Routine blood samples taken for this study are part of standard clinical care and will be processed in local laboratories for clinical purposes as per normal protocols. For study purposes, the results of these investigations will be documented.

### Outcomes {12}

#### Primary outcome measure

To confirm the safety and assess efficacy of the NMP-defatting protocol in steatotic donor livers intended for transplant.

The primary endpoint is the proportion of livers that achieve all of the following functional criteria at 6 h of perfusion [15, 17], as defined by:

- Clearance of lactate to a level < 2.5 mmol/L
- Perfusate pH ≥ 7.20
- Evidence of glucose metabolism (spontaneous fall in perfusate glucose)
- Minimum bile pH ≥ 7.5 (if bile produced)
- Bile glucose concentration ≤3 mmol/L or ≥10 mmol less than perfusate glucose
- Hepatic arterial flow ≥100 mL/min; portal venous flow ≥ 500 mL/min
- Perfusate alanine aminotransferase (ALT) < 6000 U/L at 6 h

These objective criteria, reflecting hepatic metabolism and injury, have been derived by a process of consensus amongst current NMP users. These parameters are increasingly recognised as a way to discriminate livers with favourable post-transplant outcomes and will be measured at baseline (pre-NMP) and throughout perfusion (1, 2, 4 and 6 h and end of perfusion) [15, 17]. Lactate measurements will also be taken at baseline (pre-NMP) and 5 min after start of NMP.

These functional criteria are not intended as an instruction to the implanting surgeon, but rather as a consistent endpoint for the trial. The decision as to whether a liver is actually transplanted will remain with the implanting surgeon, who will base this on a number of criteria, including some that are recipient-related rather than donor organ-related (e.g. the urgency with which the patient needs a transplant may determine the decision).

#### Secondary outcome measures

Clinical

1. Proportion of livers transplanted in the two arms
2. LiMAx (maximum liver function capacity) test performed after 1 h of liver stabilisation during NMP, repeated at 5 h and subsequently every 6 h till end of perfusion where feasible. If the decision to transplant has been made by 6 h of NMP, the test will not be repeated. The LiMAx test will allow real-time monitoring of CYP1A2 (prominent in functional livers cells and less prominent in damaged cells) and is based on the metabolism of ^13^C-methacetin. This will enable measurement of liver capacity and functional reserve during perfusion [60].
3. Cell-free DNA (cfDNA) measured at baseline (pre-NMP) and during preservation (1, 2, 4 and 6 h and end of perfusion). Further measurements taken from the recipient peri-operatively (before transplant) and re-perfusion (following liver transplantation). Post-operative samples collected on days 1, 3, 7 and 14 (if the patient is discharged prior to day 14—a sample will be collected on the day of discharge instead). Outpatient sample collection will align with clinic visits on day 30 and months 3 and 6. cfDNA has been correlated with allograft injury, rejection and formation of de novo donor-specific antibodies [61].
4. Biochemical liver function in the first 7 days post-transplant: ALT, GGT, INR, bilirubin and peak serum AST (where AST measurements available) in the first 7 days post-transplant. Peak serum AST is a validated surrogate marker, predictive of PNF as well as graft and patient survival [62]. It is also associated with histological evidence of moderate to severe reperfusion injury [63].
5. Model of Early Allograft Function (MEAF) [64]: a score (between 0 and 10) based on bilirubin, INR and ALT within the first three post-operative days.
6. Primary non-function (PNF): irreversible graft dysfunction requiring emergency liver replacement during the first 10 days after liver transplantation, in the absence of technical or immunological causes.
7. Post-reperfusion syndrome (PRS) [65]: a decrease in mean arterial pressure (MAP) of more than 30% for more than 1 min during the first 5 min after reperfusion [65].
8. Need for renal replacement therapy (haemodialysis, haemofiltration, peritoneal dialysis) during the first 7 days post-operatively.
9. Duration of ITU/HDU and hospital stay.
10. Graft survival (defined as a functioning transplant in the absence of death and re-transplantation) at day 7, day 30, month 3 and month 6.
11. Patient survival at day 7, day 30 and months 3 and month 6. In addition, 12-month clinical outcomes (obtained from NHSBT registry) will also be reported:
  a Graft and patient survival
  b Total number of days in hospital in the last year (excluding transplant admission)
  c Total number of re-admissions for:
    • Recipient infection
    • Acute rejection
    • Chronic rejection
    • Biliary complications
    • Vascular complications
    • Disease recurrence
    • Other reasons
  d Transplant-related renal dysfunction
  e Biochemistry (liver and renal function)
12. The following safety information will be recorded:
  a Organ discard rate
  b Perfusate culture. At the end of preservation, a sample will be taken for microbiological culture.
  c Adverse event rates and severity, graded according to the Clavien–Dindo classification [66] during the first 7 days, day 30, month 3 and month 6:
    • Recipient infection
    • Biopsy proven acute rejection
    • Biliary complications (biliary strictures—anastomotic and non-anastomotic, bile duct leaks)
    • Vascular complications (bleeding, hepatic artery stenosis, hepatic artery thrombosis, portal vein thrombosis)
    • Reoperation rate
  d Technical complications/device failures

Histological and biochemical

1. Correlation of pre-perfusion donor biopsy (histopathologist’s steatosis report) with:
  a WC, BMI and clinical risk scores such as the fatty liver index (FLI) and hepatic steatosis index (HSI) [67, 68] where relevant data available
  b Surgeon’s assessment [57]
  c Non-invasive pocket-sized micro-spectrometer reading. The device has been developed by SCIO - Consumer Physics (http://www.consumer-physics.com) and is CE marked. It utilises spectroscopy (absorption of near infrared light, 700–1100 nm). The commercially available device is able to quantify composition of foods, estimate body fat levels and identify analgesic agents [69]. Readings will be taken sequentially over perfusion (where feasible): at baseline (pre-NMP) and during preservation (1 and 6 h and end of perfusion) to facilitate correlation with post-perfusion (end-NMP) biopsy in addition to the pre-perfusion biopsy.
2. Histological and biochemical evidence of ischaemia–reperfusion injury (IRI):
  a Histology (formalin fixed paraffin embedded) [33]:
    (i) Neutrophil infiltration and leucocytosis determined using haematoxylin and eosin (H&E) stain
    (ii) Glycogen depletion determined using periodic acid-Schiff (PAS) stain
    (iii) Lipid peroxidation determined using 4-HNE (4-hydroxynonenal) stain

Biopsy samples collected pre-perfusion, post-perfusion and re-perfusion in the recipient (following liver transplantation).

b Cytokine profile  implicated in liver transplantation including [70]: CXCl8/IL-8, IL-10, IL-2, TNF-a, IFN-γ, IL-13, IL-4, IL-1β, IL-17A and IL-6.

Blood samples collected peri-operatively (before transplant) and re-perfusion in the recipient (following liver transplantation).

3. Histological and biochemical evidence of bile duct injury (BDI) and biliary viability [71] such as:
  a Histology (formalin-fixed paraffin embedded) biopsy samples pre-perfusion, post-perfusion and following re-perfusion in the recipient (following liver transplantation). For example, evidence of stromal necrosis, loss/injury to peribiliary glands and vascular lesions. Bile duct biopsies will only be taken if sufficient length on the bile duct and feasible to do so.
  b Bile composition measurements (if produced and measured at 1, 2, 4 and 6 h and end of perfusion) for example: low pH and bicarbonate with high glucose and lactate dehydrogenase (LDH) as indicators of poor biliary viability.

Imaging

1. Biliary strictures (anastomotic and non-anastomotic) determined by MRI scan at month 6 (±1 month) depending on site capacity using MRCP^+^ (a CE-marked advanced biliary visualisation software by Perspectum Diagnostics) [72].
2. Graft hepatic steatosis determined using multiparametric liver MRI (proton density fat fraction, cT1 and T2* mapping) at 6 (± 1 month) depending on site capacity. MRI proton density fat fraction (MRI-PDFF) has demonstrated high diagnostic accuracy in both the detection and grading of hepatic steatosis with histology as a reference standard [73, 74]. CE-marked software such as Liver*MultiScan*^TM^ developed by Perspectum Diagnostics will aide in the quantification of steatosis.

#### Mechanistic studies outcome measures

The identification of novel markers could augment current practice by predicting the outcome of each liver with objectivity. The mechanistic studies will test hypotheses based on previous published studies that have investigated markers in the field of NMP and will inform development of functional criteria and optimisation of future defatting protocols:

1. To measure the effect of the intervention on the histological degree of steatosis:
  Histological quantification of MaS measured pre-perfusion, post-perfusion and during re-perfusion in the recipient. We hypothesise that the intervention of defatting will reduce the degree of MaS and severity of NAFLD activity score [75].
2. To measure the effect of the intervention on markers on of hepatic lipid metabolism:
  Perfusate TG, insulin, ketone bodies and cytokines associated with IRI will be measured at baseline (pre-NMP) and during preservation (1, 2, 4 and 6 h and end of perfusion). Further measurements taken from the recipient peri-operatively (before transplant) and re-perfusion (following liver transplantation). This will provide insight into changes in intrahepatic lipid handling and inflammation. We hypothesise that the intervention of defatting will ‘repartition’ intrahepatic fatty acids away from esterification into oxidation pathways leading to a decrease in IHTG and a decrease in IRI-associated cytokine production [70].
  Perfusate FGF-21 will be measured at baseline (pre-NMP) and during preservation (1, 2, 4 and 6 h and end of perfusion). Further measurements taken from the recipient peri-operatively (before transplant) and re-perfusion (following liver transplantation). FGF-21 is a hormone produced in the liver involved in energy homeostasis; its secretion is attributed to metabolic stress. There is evidence that serum FGF-21 is a useful marker for steatosis and correlates with increasing steatosis grade [76]. We hypothesise that the defatting intervention will reduce FGF-21.
3. To understand the structural, cellular and metabolic effects of defatting on steatotic livers.
  Genomic analysis of samples taken pre-perfusion, post-perfusion and following re-perfusion in the recipient:
    ◦ Transcriptomics: a complex signalling cascade regulates metabolic processes within the liver. To understand the effect of NMP and the defatting intervention, genomic analysis of liver tissue will be undertaken. We hypothesise that the defatting intervention will lead to downregulation in pathways related to fat synthesis and inflammation and an upregulation in pathways related to fat disposal. Samples from livers will undergo RNA sequencing of the liver and this will be correlated with clinical outcomes. Changes in gene expression will be mapped with changes occurring in biological pathways, inferring biological changes during NMP.
  Proteomic and glycomic analysis of perfusate samples taken at baseline (pre-NMP) and during preservation. Further measurements taken from the recipient peri-operatively (before transplant) and re-perfusion (following liver transplantation):
    ◦ Proteomics: a recent study investigating the use of NMP to increase utilisation of high-risk donor livers, identified protein clusters that were able to discriminate between transplantable and non-transplantable livers (22 out of 31) as well as markers predictive of post-transplant complications [77]. We aim to determine the effect of the intervention on protein expression associated with hepatic steatosis, inflammation and IRI.
    ◦ Glycomics: the liver perfusate glycome profile may form part of future functional criteria [78]. A recent study found that the abundance of a single glycan, agalacto core-alpha-1,6-fucosylated biantennary glycan (NGA2F) was significantly higher in the perfusate of livers that developed PNF. We will test this hypothesis in sequential perfusate samples.

### Participant timeline {13}

**Fig. 6:**
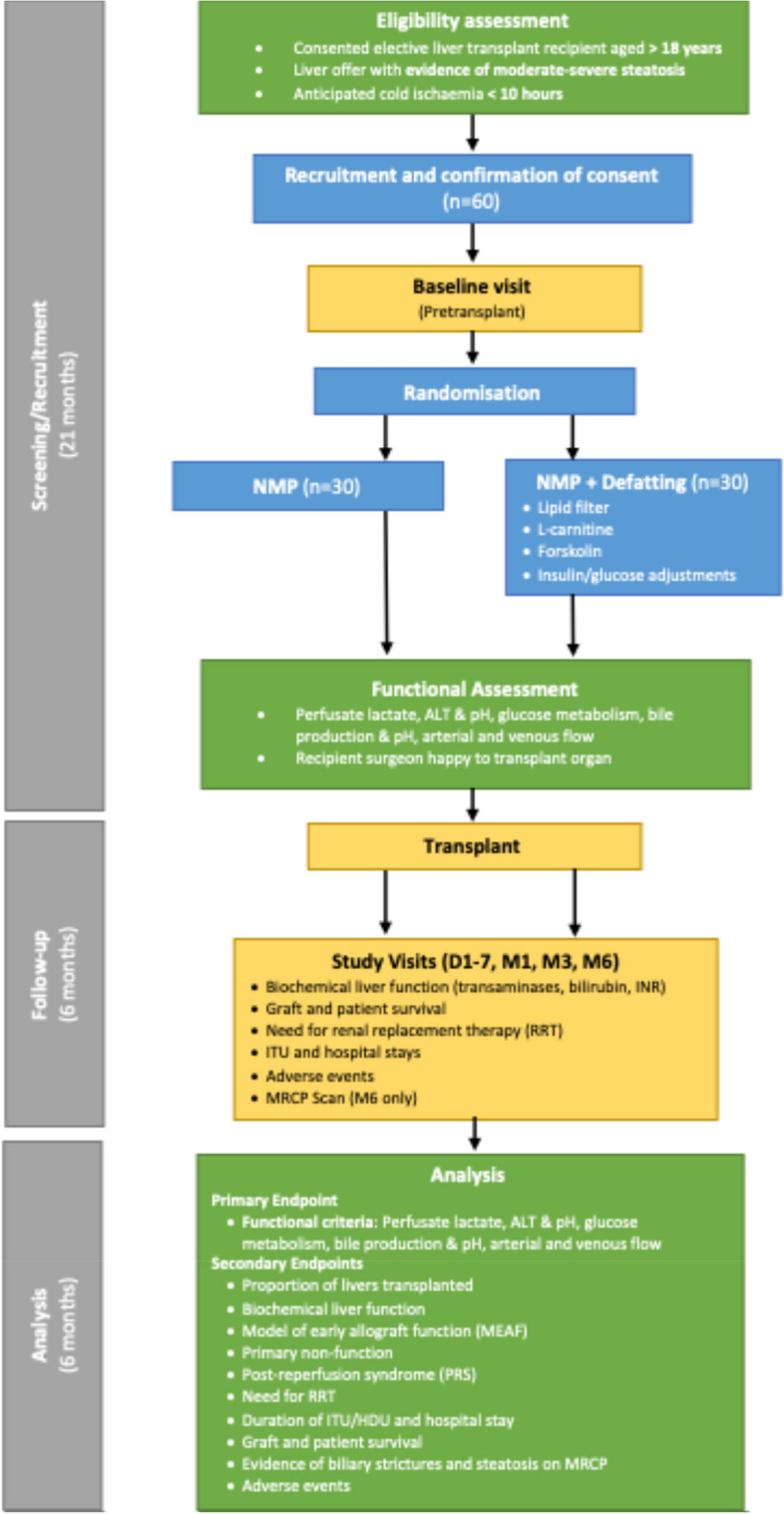
Flow of participants through the study. NMP normothermic machine perfusion, MRCP magnetic resonance cholangiopancreatography, ALT alanine transaminase

The proposed study duration is 56 months (01/04/2021 – 30/11/2025): The DeFat study set-up tasks commenced in April 2021 and the study opened for recruitment on the 23rd of February 2023. The anticipated end date for recruitment is the 30th of November 2024 (total recruitment period of 21 months) with 6 month follow-up of the last participant and 6 months analysis/dissemination (close-out) anticipated to be completed by the 30th November 2025. The flow of participants in the study is summarised in Fig. 6. In addition, all trial procedures are summarised in Table 3 (Standard Protocol Items: Recommendations For Interventional Trials, SPIRIT).

**Table 3:** SPIRIT table of enrolment, interventions, and assessments (schedule of procedures)

**Activity**	**Pre-study screening**	**Pre-study baseline**	**Pre-perfusion**	**During and end of perfusion**	**Pre-transplant**	**Post-reperfusion**	**Postoperative**									**Follow-up and close-out**			
							D1	D2	D3	D4	D5	D6	D7	D10	D14	D30	M3	M6	Close-out
**Informed consent**	X																		
**Meets inclusion/exclusion criteria**	X																		
**Randomisation**		X																	
**Donor and recipient demographics**		X																	
**Perfusion parameters and samples**			X	X	X	X													
**cfDNA samples**			X	X	X	X	X		X				X		X	X	X	X	
**Surgical variables**						X													
**Serum ALT and AST**							X	X	X	X	X	X	X			X	X	X	X
**Serum bilirubin**							X	X	X	X	X	X	X			X	X	X	X
**Serum GGT**							X	X	X	X	X	X	X			X	X	X	X
**INR**							X	X	X	X	X	X	X			X	X	X	X
Serum lactate*							X	X	X	X	X	X	X						
**Primary non-function**														X					
**Graft survival**							X	X	X	X	X	X	X	X		X	X	X	X
**Patient survival**							X	X	X	X	X	X	X	X		X	X	X	X
**Resource use**							X	X	X	X	X	X	X	X		X	X	X	X
**Safety outcomes**						X	X	X	X	X	X	X	X	X		X	X	X	X
**MRI (depending on site capacity)**																		X	

#### Baseline donor and recipient assessment

Baseline donor assessments will include donor demographics and blood test results. The following information will be recorded: age, sex, ethnicity, co-morbidities, cause of death, height, weight, BMI, WC, smoking history and alcohol consumption. Donor blood test will include last and peak serum aspartate transaminase (AST), alanine transaminase (ALT), gamma-glutamyl transferase (GGT) and serum bilirubin and sodium. Other donor information will include last and fasting triglyceride (TG), if available, evidence of TPN or enteral feed (if information available) and length of ITU stay.

Baseline recipient demographics will include recipient demographic and pre-transplant test results. The following information will be recorded: age, sex, ethnicity, co-morbidities, at the time of transplant (minimum 24 h of continuous renal replacement therapy) or two haemodialysis sessions in the previous week, aetiology of primary liver disease and primary indication of transplant, height, weight, BMI, WC (where available) and smoking history. Pre-transplant recipient blood test will include INR, creatinine, bilirubin and sodium.

#### Donor timings

These are all routinely collected at the time of retrieval and will be obtained from the NHSBT database.

The parameters to be recorded include:

- Timings:
  - ◦ Withdrawal of support (DCD donors only)
  - ◦ Onset of functional warm ischaemia (DCD donors only)
  - ◦ Cessation of donor circulation (cross clamp or asystole in DCD donors)
  - ◦ Start of cold perfusion
  - ◦ Liver removal and placement on ice
- Perfusion solution used for aortic perfusion
- Perfusion solution used for storage and transport
- Degree of steatosis (graded mild, moderate, severe)—surgeon’s assessment
- Quality of in situ perfusion (graded poor, moderate, good)

#### Preservation parameters

In addition to timings, a number of other preservation parameters will be recorded. These will include:

- Time of initiation of normothermic machine preservation
- Time of cessation of normothermic machine preservation (end flush)
- Flush solution (UW, HTK, other)
- Perfusion parameters (for NMP livers; logged automatically by the device):
  - ◦Arterial and caval pressures (in mmHg)
  - ◦ Arterial, portal and caval flow rates (in L/min)
  - ◦ pO_2_, pCO_2_ and pH
  - ◦ Blood temperature (°C), glucose (mmol/L) and bile production (mL/h)
- Perfusate biochemistry
  - ◦ Perfusate lactate at baseline (pre-NMP), 5 min after start of NMP and during preservation (1, 2, 4 and 6 h and the end of NMP)
  - ◦ Perfusate pH at baseline (pre-NMP) and during preservation (1, 2, 4 and 6 h and end of NMP)
  - ◦ Perfusate ALT at baseline (pre-NMP) and during preservation (1, 2, 4 and 6 h and end of NMP)
  - ◦ Glucose levels at baseline (pre-NMP) and during preservation (1, 2, 4 and 6 h and at the end of NMP)
  - ◦ Bile pH, glucose and bicarbonate (if bile produced) at 1, 2, 4 and 6 h and at the end of NMP
  - ◦ Bicarbonate use (time and dose of each bolus)
- Sampling:
  - ◦ Perfusate at baseline (pre-NMP) and during preservation (1, 2, 4 and 6 h and end of NMP)
  - ◦ Liver core biopsies taken pre-perfusion and post-perfusion
  - ◦ Bile duct biopsies taken pre-perfusion and post-perfusion where feasible, i.e. if sufficient bile duct length

At the end of preservation, a sample of perfusate/storage solution will be taken for microbiological culture as per standard practice.

Operative parameters

These will include:

- Total operative time: defined as time from knife-to-skin to skin closure
- Time of flush at end of NMP
- Time of liver in body (start of anastomosis)
- Time of reperfusion (portal or arterial, whichever occurs first)
- Portal reperfusion time
- Arterial reperfusion time
- Intraoperative transfusion of blood products measured in units
- The use of veno-venous bypass or porto-caval shunts
- Type of caval anastomosis (standard end-end, piggyback (end-side or side-side))

#### Intra-operative outcome assessment

Recipient blood samples (before and after transplant) and post-reperfusion liver biopsy (as well as bile duct biopsy where feasible) will be taken:

- ◦ Peri-operative blood taken (before transplant) and re-perfusion (following liver transplantation)
- ◦ Liver core biopsy taken post-reperfusion
- ◦ Bile duct biopsy taken post-reperfusion where feasible i.e. if sufficient bile duct length

These samples will be taken to determine the severity of ischaemia–reperfusion injury and changes in mean arterial pressure will be recorded to assess for post-reperfusion syndrome:

- Histological and biochemical evidence of ischaemia–reperfusion injury (IRI) in recipient [70]:
- Cytokine profile implicated in liver transplantation including [70]: CXCl8/IL-8, IL-10, IL-2, TNF-a, IFN-γ, IL-13, IL-4, IL-1β, IL-17A and IL-6.
- Liver histology (formalin fixed paraffin embedded biopsy) [33]:
- Neutrophil infiltration and leucocytosis determined using haematoxylin and eosin (H&E) stain
- Glycogen depletion determined using periodic acid-Schiff (PAS) stain
- Lipid peroxidation determined using 4-HNE (4-hydroxynonenal) stain
- Bile duct histology (formalin fixed paraffin embedded biopsy) [71]:
- Evidence of stroma necrosis, extramural peribiliary glands (loss/injury to cells) and presence of vascular lesions
- Post-reperfusion syndrome (PRS) [65], a decrease in mean arterial pressure (MAP) of more than 30% for more than 1 min during the first 5 min after reperfusion [65].

#### Declines and discards

If a decision is made to decline an organ at any point after retrieval (but before randomisation), any donor and preservation data recorded will be kept, and the reason for decline clearly documented in the eCRF. If deemed appropriate by the OTDT Hub, the organ may be offered to other centres on the matching run with liver allocation as per standard of care. If the liver is already on the OrganOx *metra*^*®*^ device, every attempt should be made to keep it on the device (as per national agreement). If all centres subsequently decline an organ, the organ will be documented as a discard and it will be offered for research or disposed of as per standard procedures.

Data will also be collected for all discards (including reason for discard) from point of randomisation.

#### Inpatient stay

Patients will be assessed daily by their clinical team and managed according to standard care protocols at the site with clinical information obtained from medical records.

Post-operative outcome assessment

The investigations performed form part of routine clinical care.

The following biochemical outcomes will be recorded:

- Daily serum samples for the first 7 days post-transplant, to include:
  - ◦ Serum bilirubin (measured in μmol/l)
  - ◦ Serum gamma-glutamyl transferase (GGT; measured in IU/L)
  - ◦ Serum aspartate transaminase (AST; measured in IU/L) or serum alanine transaminase (ALT; measured in IU/L) depending on liver transplant centre
  - ◦ International normalised ratio (INR)
  - ◦ Serum alkaline phosphatase (ALP; measured in IU/L)
  - ◦ Blood urea (mmol/L)
  - ◦ Serum creatinine (μmol/L)
- Daily serum lactate (measured in mmol/L) whilst on ITU/HDU.

The first measurements should be taken at 12 to 24 (±6 h) hours post-transplant. For subsequent measurements, in the event that more than one measurement is taken in a 24-h period, the measurement taken closest to the specified follow-up time-point should be used.

- cfDNA measurements perioperatively in the recipient (before and after transplant) and on days 1, 3, 7 and 14 (if the patient is discharged prior to day 14—a sample will be collected on the day of discharge instead). cfDNA has been correlated with allograft injury, rejection and formation of de novo donor specific antibodies [61]. These research samples will be taken alongside routine clinical samples in order to minimise any additional tests.
- Model of Early Allograft Function (MEAF) [64]: a score (between 0 and 10) based on bilirubin, INR and ALT within the first three post-operative days.

Other outcomes to be recorded include:

- Length of stay in level 2/level 3 care (ITU/HDU) (days)
- Total length of hospital stay (days)
- Requirement for renal replacement therapy during transplant admission (haemodialysis (HD), haemodiafiltration (HDF), haemofiltration (HF), peritoneal dialysis (PFD))
- Graft and patient survival at day 7 post-transplant
- Primary non-function: irreversible graft dysfunction requiring emergency liver replacement during the first 10 days after liver transplantation, in the absence of technical or immunological causes.

Safety outcomes

- Recipient infection (defined as both clinically diagnosed treated infection and infection with a positive microbiological culture result)
- Clinically suspected treated rejection
- Biopsy-proven acute rejection episodes
- Biliary complications diagnosed radiologically, e.g. a non-protocol MRI or CT scan in clinically symptomatic patient:
  ◦ Biliary strictures——anastomotic and non-anastomotic. Defined as those requiring surgical or radiological intervention
  ◦ Bile duct leaks. Defined as those requiring drainage, refashioning of anastomosis or stenting.
- Vascular complications
  ◦ Bleeding. Defined as bleeding requiring transfusion and/or radiological/surgical intervention.
  ◦ Hepatic artery stenosis. Defined as causing graft dysfunction requiring radiological or surgical intervention or resulting in graft loss.
  ◦ Hepatic artery thrombosis. Defined as formation of new clot resulting in graft dysfunction or loss, or requiring pharmacological, radiological or surgical intervention.
  ◦ Portal vein thrombosis. Defined as formation of new clot resulting in graft dysfunction or loss, or requiring pharmacological, radiological or surgical intervention.
  ◦ Portal vein stenosis. Defined as causing graft dysfunction requiring radiological or surgical intervention or resulting in graft loss.
  ◦ IVC/hepatic vein occlusion. Defined as formation of new clot resulting in graft dysfunction or loss, or requiring pharmacological, radiological or surgical intervention.
- Reoperation rate
- Technical complications and device failures
- Any other reported adverse event

Severity will be graded according to the Clavien–Dindo classification [66]—*Supplemental *Table 1.

Immunosuppression

Details of induction immunosuppression and maintenance immunosuppression (including doses) at day 7 post-transplant will be recorded.

#### Subsequent study visits and later outcomes

Subsequent study visits, where possible, will coincide with routine outpatient appointments. If the recipient is an inpatient, assessment will be made in hospital where appropriate. The study visits will occur on the following dates:

- Study visit 2—Day 30 (± 2 weeks)
- Study visit 3—Month 3 (± 1 month)
- Study visit 4—Month 6 (± 1 month)

The outcome assessments for each study visit are summarised in Table 4.

**Table 4:** Study outcome measures and participant visits

Endpoint measure	**Inpatient stay** **Study visit 1**	**Day 30 (± 2 weeks)** **Study visit 2**	**Month 3 (± 1 month)** **Study visit 3**	**Month 6 (± 1 month)** **Study visit 4**
Biochemical	Days 1–7: • Bilirubin (μmol/L) • GGT (IU/L) • AST (IU/L)/ALT (IU/L) • INR • ALP (IU/L) • Urea (mmol/L) • Creatinine (μmol/L) • Lactate (HDU/ITU) Days 1–3: • MEAF score	• Bilirubin (μmol/L) • GGT (IU/L) • AST (IU/L)/ALT (IU/L) • INR • ALP (IU/L) • Urea (mmol/L) • Creatinine (μmol/L)	• Bilirubin (μmol/L) • GGT (IU/L) • AST (IU/L)/ALT (IU/L) • INR • ALP (IU/L) • Urea (mmol/L) • Creatinine (μmol/L)	• Bilirubin (μmol/L) • GGT (IU/L) • AST (IU/L)/ALT (IU/L) • INR • ALP (IU/L) • Urea (mmol/L) • Creatinine (μmol/L)
cfDNA	Days 1, 3, 7, 14 or date of discharge	Sample to be taken with routine bloods	Sample to be taken with routine bloods	Sample to be taken with routine bloods
Peri-operative and survival	• Length of stay in (ITU/HDU) (days) • Total length of hospital stay (days) • Requirement for renal replacement therapy • Graft and patient survival at day 7 post-transplant • Primary non-function	• Graft and patient survival at day 30 post-transplant • Requirement for renal replacement therapy (HD, HF, HDF, PD) at any time	• Graft and patient survival at month 3 post-transplant • Requirement for renal replacement therapy (HD, HF, HDF, PD) at any time	• Graft and patient survival at month 6 post-transplant • Protocol MRI Scan (depending on site capacity) • Requirement for renal replacement therapy (HD, HF, HDF, PD) at any time
Safety	• Recipient infection^a^ • Clinically suspected treated rejection • Biopsy-proven acute rejection • Biliary complications^b (i-ii)^ • Vascular complications^c (i-vi)^ • Reoperation rate • Technical complications and device failures • Any other reported adverse event	• Recipient infection^a^ • Biopsy-proven acute rejection • Biliary complications^b (i-ii)^ • Vascular complications^c (i-vi)^ • Reoperation rate • Technical complications and device failures • Any other reported adverse event	• Recipient infection (CMV infection, fungal infection, post-operative sepsis) • Biopsy-proven acute rejection episodes • Biliary complications^b (i-ii)^ • Vascular complications^c (i-vi)^ • Reoperation rate • Technical complications and device failures • Any other reported adverse event	• Recipient infection (CMV infection, fungal infection, post-operative sepsis) • Biopsy-proven acute rejection episodes • Biliary complications^b (i)^ • Vascular complications^c (i-vi)^ • Reoperation rate • Technical complications and device failures • Any other reported adverse event
Immunosuppression	Immunosuppression regimen at day 7 (including doses)	Immunosuppression regimen at day 30 (including doses)	Immunosuppression regimen at month 3 (including doses)	Immunosuppression regimen at month 6 (including doses)

^a^Infection, defined as both clinically diagnosed treated infection and infection with a positive microbiological culture result

^b^Biliary complications diagnosed radiologically, e.g. a non-protocol MRI or CT scan in clinically symptomatic patient: (i) biliary strictures—anastomotic and non-anastomotic. Defined as those requiring surgical or radiological intervention and (ii) bile duct leaks. Defined as those requiring drainage, refashioning of anastomosis or stenting

^c^Vascular complications including (i) bleeding, defined as bleeding requiring transfusion and/or radiological/surgical intervention; (ii) hepatic artery stenosis, defined as causing graft dysfunction requiring radiological or surgical intervention or resulting in graft loss; (iii) hepatic artery thrombosis, defined as formation of new clot resulting in graft dysfunction or loss, or requiring pharmacological, radiological or surgical intervention; (iv) portal vein thrombosis, defined as formation of new clot resulting in graft dysfunction or loss, or requiring pharmacological, radiological or surgical intervention, (v) portal vein stenosis, defined as causing graft dysfunction requiring radiological or surgical intervention or resulting in graft loss and (vi) IVC/hepatic vein occlusion, defined as formation of new clot resulting in graft dysfunction or loss, or requiring pharmacological, radiological or surgical intervention

Whilst the endpoint for trial participation will be 6 months, patients will also be consented for ongoing follow-up (12 months) by linkage to outcomes recorded by in the NHSBT transplant registry. This will allow the ongoing assessment of resource use (hospital stay and reasons for re-admission), biochemistry results (liver and renal function), transplant-related renal dysfunction and longer-term patient/graft survival.

### Sample size {14}

Our preliminary data described above showed that 40% more livers met functional criteria for transplantation where NMP was combined with defatting versus NMP alone (100% vs.60%) [33]. However, this is based on a small sample size and the interventions were tested on a very high-risk group of livers that had all been previously discarded. Using the proposed inclusion criteria, a smaller effect size is anticipated. Whilst the present study is not primarily intended to demonstrate efficacy, a sample size of 60 livers (30 per group) will provide greater than 80% power to detect a difference of 30% (from 65% in the control NMP arm) in those meeting criteria for transplantation (at 5% significance): this is a clinically significant outcome. This sample size should provide sufficient information for the design of a larger, phase III study to formally test the efficacy of the intervention.

### Recruitment {15}

The annual NHSBT report (2018–19) shows that of 735 adult elective liver transplants, 618 (84%) were performed at the participating liver transplant centres [11]. Data from within Eurotransplant show that 23% of livers have moderate to severe steatosis (>30%) on histology [79]. This predicts that 142 livers (annually) and 213 livers (over 18 months) with moderate to severe steatosis would be available at the centres participating in this study. Allowing for a 50% recruitment rate, the recruitment of 60 livers in 18 months is feasible (allowing for small proportion non-steatotic livers to be randomised).

## Assignment of interventions: allocation

### Sequence generation {16a}

If the liver is eligible for the study (at the point of organ inspection by a surgeon from the implanting team at the liver transplant centre) or with results of a clinical biopsy, randomisation will be conducted by the trial co-ordinator (clinical research fellow) who will deliver and be unblinded to the intervention.

Once eligibility is confirmed, the central trial team will use an on-line randomisation service (sealedenvelope.com) to allocate the liver to NMP or NMP with defatting interventions. The allocation sequence will be produced by sealed envelope and quality checked by the trial statistician.

### Concealment mechanism {16b}

Donor organs meeting enrolment criteria will be randomised, using a 1:1 allocation ratio, using permuted blocks of varying undisclosed size and will be stratified by donor organ type (DCD/DBD). The randomisation list will only be accessible to the trial statisticians and sealed envelope. Randomised livers that are not perfused due to unforeseen reasons will not be replaced. It is anticipated that non-perfusion of a randomised liver will be a very uncommon event.

### Implementation {16c}

This is a single-blinded randomised clinical trial. Perfusions will be performed by a member of the central trial team. The trial co-ordinator (clinical research fellow) and/or member of the central trial team will be responsible for randomisation and will be unblinded to the intervention.

## Assignment of interventions: blinding

### Who will be blinded {17a}

After randomisation, setting up the NMP device will follow standard practice, with addition of the apheresis filter and pharmacological protocol. The presence or absence of the lipoprotein apheresis filter will be concealed through the use of a ‘dummy’ filter covered by a drape. This will prevent the local transplant and research team (and therefore the patient) from knowing the study allocation. In the case of a medical emergency or safety concern ascribed to the perfusion, rapid identification of the trial treatment and randomisation code will be permitted and documented.

### Procedure for unblinding if needed {17b}

If urgent unblinding is considered necessary during the liver perfusion, the trial co-ordinator (clinical research fellow) or a member of the central trial team who will be co-ordinating the perfusion and therefore aware of the liver randomisation will disclose allocation to the clinical team. In other circumstances, the PI (or assigned deputy) will request and be given access to the unblinding facility for the individual randomisation through the web-based service (www.sealedenvelope.com). Unblinding of randomisations will be documented along with the reasons triggering them. The individual requesting unblinding at the site will receive an automatic email notification with the arm allocation of the perfused liver. All instances of unblinding should be reported to NHSBT CTU and chief investigator (CI) as soon as possible. Details of the randomisation process and emergency code breaking will be located in the site file. The trial co-ordinator (clinical research fellow) will not be involved in any clinical decision making or any of the study assessments.

## Data collection and management

### Plans for assessment and collection of outcomes {18a}

A detailed data management plan will be developed to outline the data management processing, data cleaning and QC procedures for the trial. The data management aspects of the study are summarised here:

#### Source data

Source documents are where data are first recorded and from which participants’ CRF data are obtained. These include, but are not limited to, hospital records (from which medical history and previous and concurrent medication may be summarised into the CRF), clinical and office charts, laboratory and pharmacy records, diaries, microfiches, radiographs, and correspondence.

CRF entries will be considered source data if the CRF is the site of the original recording (e.g. there is no other written or electronic record of data). All documents will be stored safely in confidential conditions. On all trial-specific documents, other than the signed consent, the participant will be referred to by the patient trial ID, not by name.

#### Monitoring

Regular monitoring will be performed according to the trial specific monitoring plan. Data will be evaluated for compliance with the protocol and accuracy in relation to source documents as these are defined in the trial specific monitoring plan. Following written standard operating procedures, the monitors will verify that the clinical trial is conducted and data are generated, documented and reported in compliance with the protocol, GCP and the applicable regulatory requirements.

#### Local investigator and site personnel training

All key site personnel must undergo relevant training in advance of the site initiation in accordance with Good Clinical Practice (GCP) guidelines. Such training will be documented. In addition, training for site staff will be provided by OrganOx Ltd. in advance of recruitment of the first patient. A record of all device training will be maintained. All personnel involved in randomisation and data entry will also be trained in the use of the online randomisation and data collection tool by members of the clinical trials unit, and records of such training will be maintained.

#### Study documentation

It is the responsibility of the local investigator to maintain complete, accurate and current study records. Each investigator will be provided with an investigator site file, online access to the case reporting system and other associated study specific documentation by the co-ordinating centre. Such records will be maintained during the course of the study and for up to 5 years following the date on which the study is terminated or completed, in accordance with local regulatory requirements.

### Plans to promote participant retention and complete follow-up {18b}

The follow-up visits for the DeFat study align with routine clinical visits. All patients completing the 6-month follow-up assessment will be regarded as having completed the primary study. All patients will be encouraged to complete study follow-up, and all reasonable efforts will be made to ensure completeness of follow-up. Measures include ensuring that sample collection and assessments are made, where possible, at routine hospital visits rather than additional appointments and that patients do not incur extra financial costs (e.g. travelling costs) as a result of study participation.

It is understood that study participants may withdraw consent for study participation at any time irrespective of their reasons. The investigators may also withdraw a recipient from the study in order to protect their safety and/or if they are unwilling or unable to comply with the required study procedures. We will keep all data accrued to the point of withdrawal, as is stipulated in the trial consent form.

Possible reasons for investigator-led withdrawal of a participant from the trial include:

- Major protocol deviation
- Withdrawal of consent
- Loss to follow-up
- SAE/SUSAR
- Early termination of study

In the event of a patient withdrawing from the trial, the reason for withdrawal must be documented on the eCRF. Such patients will be asked whether they consent to the use of ongoing data collected as standard in the national transplant registry for the purposes of this study.

### Data management {19}

#### Data recording and record keeping

Randomised liver and participant data will be entered onto the trial database designed and administered by the NHSBT CTU data management team using MACRO™, a commercially available FDA 21 Code of Federal Regulations (CFR) Part 11 compliant clinical trial database system produced by InferMed. Following completion of analysis, the trial database will be archived in accordance with NHSBT’s policies.

The study team must keep the signed informed consent forms, all trial documentation and source documents collected during the trial in a secure location (e.g. locked filing cabinets in a room with restricted access). All data must be accessible to the competent authorities and the sponsor with suitable notice for inspection.

The participants will be identified by a unique patient trial ID in any database. Participant identifiers (e.g. NHS number) will only be stored where required for linkage to external data sources (e.g. NHSBT). Individual participants will not be identified in the resulting publications and presentations from the trial. This trial will comply with the UK Data Protection Act (2018) and the General Data Protection Regulation

All trial documentation must be retained for at least 5 years after trial completion or termination. In addition, the investigator must not discard or destroy any trial specific materials unless otherwise instructed by NHSBT.

#### Use of registry data

The UK Transplant Registry will be the primary source of data about resource use (hospital stay and reasons for re-admission), biochemistry results (liver and renal function), transplant-related renal dysfunction and graft/patient survival at 12 months. Where available, the primary source of recipient outcome data will be that collected from the electronic case report forms. Where primary or secondary outcome data are missing, we will attempt to link to the NHS Blood and Transplant registry to obtain missing data where recorded. The primary source for 12 months outcome data will be the UK Transplant Registry. Linkage between trial and registry data will only be undertaken by statisticians working on the trial and registry identifiers will be removed from datasets after linkage has been undertaken. NHS Blood and Transplant Information Governance have conducted a Data Protection Impact Assessment, are satisfied that confidentiality and data protection measures are in place and approved the use of UK Transplant Registry Data for this study.

### Confidentiality {27}

The study will comply with the General Data Protection Regulation (GDPR) and Data Protection Act 2018, which require data to be de-identified as soon as it is practical to do so. The processing of the personal data of both donors and recipients will be minimised by making use of unique liver and patient trial IDs only on all study documents and any electronic database(s). All documents will be stored securely and only accessible by study staff and authorised personnel. The study staff will safeguard the privacy of participants’ personal data.

### Plans for collection, laboratory evaluation and storage of biological specimens for genetic or molecular analysis in this trial/future use {33}

The trial co-ordinator (clinical research fellow) and/or member of the central trial team will be responsible for collection of perfusate and peri-operative samples.

Perfusate samples will be collected at baseline (pre-NMP) and during preservation (1, 2, 4 and 6 h and at the end of perfusion). Blood samples will also be collected peri-operatively before transplant and post-reperfusion. Three samples will be taken at each timepoint:

- 1× EDTA separator tube (or universal tube if EDTA not available)
- 1× Serum separator tube
- 1× Streck tube for measurement of cfDNA

To ensure minimal sample degradation and pre analytical variability, perfusate and peri-operative samples should be kept at room temperature prior to separation of plasma from cellular parts. Separation of cells from plasma and serum should be achieved by centrifugation at 1500*g* for 10 min at room temperature as close as possible to blood collection. After centrifugation, plasma and serum samples should be kept at 4°C. The perfusate samples will be transferred into 1.0–2.0-mL aliquots and subsequently transported to Oxford University Hospitals NHS Foundation Trust and stored frozen at −80 °C.

In addition to perfusate and peri-operative measurements, cfDNA will also be measured post-operatively. Samples will be collected from recipients in Streck tubes on days 1, 3, 7 and 14 (if discharged before day 14, a sample will be collected on date of discharge). These post-operative samples will be taken by the clinical team at each liver transplant centre and collection will align with routine clinical samples. Follow-up measurements will align with clinic visits on day 30, months 3 and 6. The Streck tubes are stable at room temperature for up to 7 days and will be shipped to an accredited laboratory in the United Kingdom (UK) or abroad.

Bile (if produced) will be collected at 1, 2, 4 and 6 h and at the end of perfusion and transferred into 1.0–2.0-mL aliquots for subsequent transport to Oxford University Hospitals NHS Foundation Trust and stored frozen at −80 °C.

Liver and bile duct biopsies will be taken before perfusion, at the end of perfusion and following reperfusion in the recipient (prior to skin closure). A total of five core liver biopsies will be taken: two biopsies before perfusion, two at the end of perfusion and one following reperfusion. Each liver biopsy will be divided into two segments. Per liver, one segment will be stored in formalin and the remaining segments will be frozen. A single bile duct biopsy will be taken at each timepoint where feasible, i.e. if sufficient length on the bile duct. The bile duct biopsies will not be divided into two and only stored in formalin. The formalin samples will be stored at Oxford University Hospitals NHS Foundation Trust. Frozen samples will also be stored at this location at −80 °C.

Pre-implantation research biopsy samples will only be taken where donor family consent to research is in place. Sample collection will follow national regulations and standard operating procedures. Following collection, storage and transportation will be in accordance with the Human Tissue Authority guidelines and Trust policies. The trial team will have access to the samples and will ensure storage at Oxford University Hospitals NHS Foundation Trust. All research samples will be stored for future research and the mechanistic studies described in the study protocol.

Overall, the trial ID will be used as an identifier for all stored samples. Only personnel authorised by the chief investigator will be responsible for the storage, access and release of these samples for analysis.

## Statistical methods

### Statistical methods for primary and secondary outcomes {20a}

#### Description of statistical methods

Primary endpoint data will be presented for each arm separately, and the primary analysis will be a logistic regression model, with adjustment for donor organ type (DCD/DBD) to assess whether there is a statistically significant difference between treatment arms. An additional analysis where the model is adjusted for transplant centre will also be considered given sufficient counts within centre. The additional model will employ Firth’s penalised maximum likelihood to mitigate for small sample bias and overfitting. This analysis will include all livers randomised in an intention-to-treat (ITT) analysis. The proportion of livers actually transplanted will be presented and analysed in a similar way. A modified intention-to-treat analysis (mITT) will be considered for livers that were randomised but were not subsequently perfused. Reasons for not undergoing perfusion will be documented and an independent adjudication panel will consider inclusions to a mITT analyses after consideration of these reasons, on a case-by-case basis. There may be indirect logistical reasons rendering the inclusion of non-perfused livers to an ITT inappropriate, i.e. these reasons are not completely unrelated to the allocated intervention.

Many of the secondary endpoints are only relevant for livers that are actually transplanted, and so these analyses will be conducted on a modified intention-to-treat (mITT) population of all livers randomised and transplanted, analysed according to randomised treatment. Outcomes will be presented as counts and proportions, means and standard deviations, or medians and interquartile ranges as appropriate and analysed using linear regression for continuous outcomes; logistic regression for binary outcomes; and Cox regression analysis for time to event outcomes. In addition to the peak ALT/AST in the first 7 days post-transplant, the area under the curve will be used summarise the post-operative biochemical markers (ALT, AST, GGT, INR and bilirubin) levels over time. There will be very limited statistical testing of secondary endpoints in this small trial, and the focus will be on presenting the effect size of the defatting + NMP intervention relative to standard NMP with 95% confidence interval to help inform the design of a future definitive trial.

An ITT analysis will be performed for the primary outcome and secondary (donor liver related) outcomes. Secondary (recipient) outcomes will be analysed using a mITT approach. This analysis will exclude livers perfused but not transplanted for any reason.

For the mechanistic work, measures will be compared before and after perfusion to assess for a change during machine perfusion. A paired *t*-test will be used to compare the means of these levels pre- and post treatment to determine whether any change is statistically significant. For all analyses with statistical testing, a *p*-value of < 0.05 will be used to determine statistical significance.

Full details of the statistical analysis plan will be made available on the ISRCTN registry webpage (ISRCTN 14957538: 10.1186/ISRCTN14957538).

### Interim analyses {21b}

Data will be reviewed by the data monitoring committee (DMC) after first ten liver perfusions. If there are no safety concerns, recruitment will continue as per the study protocol.

The DMC or sponsor may recommend suspension or termination of the study either at an individual investigation site or the entire study for significant and documented reasons. An investigator and ethics committee may suspend or prematurely terminate participation in the study at the investigation sites for which they are responsible. If suspicion of an unacceptable risk to subjects arises during the study, or when so instructed by the ethics committee, the sponsor shall suspend the study whilst the risk is assessed. The sponsor shall terminate the study if an unacceptable risk is confirmed.

The sponsor shall consider terminating or suspending the participation of a particular study site or investigator in the study if monitoring or auditing identifies serious or repeated deviations on the part of an investigator.

If suspension or premature termination occurs, the terminating party shall justify its decision in writing and promptly inform the other parties with whom they are in direct communication. The chief investigator and sponsor shall keep each other informed of any communication received from either the ethics committee.

If, for any reason, the sponsor suspends or prematurely terminates the study at an individual investigation site, the sponsor shall inform the Ethics Committee, either through the chief investigator or the sponsor. If the suspension or premature termination was in the interest of safety, the sponsor shall inform all other investigators.

If suspension or premature termination occurs,

- the sponsor shall remain responsible for providing resources to fulfil the obligations from the study protocol and existing agreements for following up the subjects enrolled in the study, and
- the chief investigator or authorised designee shall promptly inform the enrolled subjects at his/her study site, if appropriate.

### Methods for additional analyses (e.g. subgroup analyses) {20b}

There are no plans for additional subgroup analyses.

### Methods in analysis to handle protocol non-adherence and any statistical methods to handle missing data {20c}

#### Procedure for accounting for missing, unused, and spurious data

Withdrawals from the trial after implantation will be documented, and a narrative analysis of withdrawals will be performed. Recipients withdrawing from the trial after implantation will be included in analysis using all available data. Consideration will be given to model-based and multiple imputation methods and detailed in the SAP. The rational for this is briefly described below.

The primary outcome will be available for all livers randomised and perfused. Missing data will be described and reported, although it is anticipated very few patients will be lost to follow-up. The reason for missingness for variables implicated in the primary analyses will be explored through regression of the missing variable indicator on other observables and detailed in the SAP. All analyses will include all data available.

The small sample size poses significant limitations for building robust multiple imputation models for handling missing data. Consideration of multiple imputation model will be given only for the analyses of selected/primary outcomes when it is valid to do so:

- The proportion of missing data is less than 5% and the impact of missing is negligible
- When no additional information can be obtained (no auxiliary variables to use for imputation can be identified)
- When missing data can be assumed to be missing completely at random from the outset
- When missingness can be assumed missing at random conditional on other observable data

In the case where the primary outcomes are not missing at random, then a ‘worst-best-case’ scenario sensitivity analyses will be undertaken to show the range of uncertainty due to missing. Briefly, in such analyses, a ‘worse-best-case’ scenario dataset will be generated where it is assumed that all participants missing the primary outcome in one group had a harmful outcome and all missing the outcome in the other group had a beneficial outcome.

### Plans to give access to the full protocol, participant level-data and statistical code {31c}

Direct access will be granted to authorised representatives from the sponsor and the host institution to permit trial-related monitoring, audits and inspections.

## Oversight and monitoring

### Composition of the coordinating centre and trial steering committee {5d}

#### Trial management group (TMG)

A TMG comprising the CI, other lead investigators, local principal investigators and members of the CTU. The TMG will be responsible for the day-to-day running and management of the trial. It will meet at least twice a year, more often during set-up and close down phases of the trial. At least one face-to-face meeting will be held each year.

#### Trial steering committee (TSC)

The role of the TSC is to:

- provide expert oversight of the trial
- maintain confidentiality of all trial information not already in the public domain
- make decisions as to the continuation of the trial
- monitor recruitment rates and advise the TMG on recruitment issues
- review and approve V1.0 of the protocol and any substantial amendments
- review regular progress reports of the trial from the trial team
- receive feedback from the DMC and consider their recommendations, including any ethical implications arising from their advice
- assess the impact and relevance of any accumulating external evidence
- monitor completion of case report forms (CRFs) and comment on strategies from TMG to deal with problems
- monitor protocol deviations and advise the TMG on remedial action
- monitor any quality issues, e.g. serious breaches and advise TMG on remedial action approve additional sub-studies
- oversee the timely reporting of trial results
- approve the statistical analysis plan
- approve the publication policy
- approve the main trial manuscript
- approve abstracts and presentations of results during the trial and on completion
- approve any requests for release of data or samples including clinical data and stored biological samples

The ultimate decision on continuation of the trial lies with the TSC.

### Composition of the data monitoring committee, its role and reporting structure {21a}

#### Safety monitoring committee

The trial has a data monitoring committee (DMC) which consists of at least three independent members, including clinicians with relevant expertise and a statistical expert, independent from the Investigators and the funding source. The DMC will periodically review accruing data to safeguard the interests of the trial participants, potential participants and future patients and assess the safety of the interventions. As a result of the reviews, the DMC may make recommendations to the TSC, including premature termination of the trial, should they feel it is indicated.

A separate DMC charter will contain full details of the committee and its roles and reporting structure.

### Adverse event reporting and harms {22}

#### Safety reporting

Adverse event definitions are provided in Table 5. The below sections describe the required reporting for adverse events within the clinical trial. This is in addition to the standard incident reporting to the device manufacturer and to clinical governance at NHSBT. It is a statutory condition of a licence for procurement or transplantation activity to rapidly report to NHSBT (acting on behalf of the HTA), relevant and necessary information concerning adverse events which may influence the quality and safety of organs. All study sites will therefore follow their usual procedures for highlighting concerns—by completing an NHSBT incident submission form: https://safe.nhsbt.nhs.uk/IncidentSubmission/Pages/IncidentSubmissionForm.aspx.

**Table 5:** Adverse event definitions

Adverse event (AE)	Any untoward medical occurrence, unintended disease or injury or untoward clinical signs (including abnormal laboratory findings) whether or not related to the study intervention.
Serious adverse event (SAE)	An adverse event that: • Led to death • Resulted in serious deterioration in the health of the subject that: ◦ resulted in a life-threatening illness or injury ◦ resulted in a permanent impairment of a body structure or a body function ◦ required in-patient care or prolongation of hospitalisation ◦ resulted in persistent or significant disability or incapacity ◦ resulted in congenital anomaly or birth defect ◦ resulted in medical or surgical intervention to prevent life-threatening illness or injury or permanent impairment to a body structure or a body function. This includes device deficiencies that might have led to a serious adverse event if: a) suitable action had not been taken or b) intervention had not been made or c) circumstances had been less fortunate

These reports will be reviewed periodically by the data monitoring committee (DMC). A safety review will be conducted by DMC after the first ten liver perfusions. All available data will be reviewed with a focus on adverse events, graft and patient survival, as well as organ utilisation.

Untoward incidents related to the process of organ retrieval and transplantation is routinely collected by NHSBT. Further detail may be found here: http://www.odt.nhs.uk/odt/governance-and-quality/incident-reporting/.

#### Severity definitions

The following definitions will be used to determine the severity rating for all adverse events:

- Mild: awareness of signs or symptoms that does not interfere with the subject’s usual activity or is transient that resolved without treatment and with no sequelae.
- Moderate: a sign or symptom, which interferes with the subject’s usual activity.
- Severe: incapacity with inability to do work or perform usual activities.

NB: to avoid confusion or misunderstanding of the difference between the terms ‘serious’ and ‘severe’, the following note of clarification is provided: ‘severe’ is often used to describe intensity of a specific event, which *may* be of relatively minor medical significance. ‘Seriousness’ is the regulatory definition supplied above.

#### Anticipated adverse events

As liver transplant recipients, all recruits to the DeFat trial are at high risk of experiencing AEs due to the complexity of their condition. Many of these events are anticipated as a result of the patient’s medical condition and standard treatment received in hospital. We will only document adverse events if in the opinion of the investigator they are likely to be associated with the trial intervention.

All adverse events meeting the definition of serious adverse event will be recorded.

#### Assessment of causality

The relationship of each adverse event to the trial procedures, conduct or intervention must be determined by a medically qualified individual according to the following definitions:

Related: the adverse event follows a reasonable temporal sequence from the trial procedures, conduct or intervention. It cannot reasonably be attributed to any other cause.

Not related: the adverse event is probably produced by the participant’s clinical state or by other modes of therapy administered to the participant.

#### Procedures for reporting adverse events

It is the responsibility of the local investigator to ensure that all adverse events considered related to the intervention and occurring during the course of the study are recorded. This will include but not be limited to:

- A description of the event
- The dates of the onset and resolution
- Action taken
- Outcome
- Assessment of relatedness to the trial procedures, conduct or intervention
- Whether the AE is serious or not

Whether the AE arises from errors in OrganOx *metra*^*®*^ device functioning or use, adverse events that occur during the course of the study should be treated by established standards of care that will protect the life and health of the study subjects.

Adverse events considered related to the intervention should be recorded on the eCRF via the MACRO database provided. If the eCRF is unavailable for any reason, a paper version of the form should be completed.

The severity of events will be assessed on the following scale: 1 = mild, 2 = moderate, 3 = severe.

Non-serious AEs, considered related to the trial procedures, conduct or intervention as judged by a medically qualified investigator or the sponsor, will be followed up until resolution.

#### Reporting procedures for serious adverse events

It is the responsibility of the local investigator to ensure that all adverse events which fall in to the category of serious adverse events (SAEs) are reported to NHSBT Clinical Trials Unit, chief investigator, central investigators and, if required, to their local R&D department as soon as possible after becoming aware of the event but no later than 24 h. This will include but not be limited to:

- A description of the event
- The dates of the onset and resolution
- Action taken
- Outcome
- Assessment of relatedness to the trial procedures, conduct or intervention

Serious adverse events will be collected from transplant until 6 months following the transplant, via a purposely designed MACRO database (access via www.ctu.nhsbt.nhs.uk/macro). SAEs will be automatically notified to NHSBT CTU. If the eCRF is unavailable for any reason, a paper version of the form should be completed, scanned and emailed to serious_adverse_events@nhsbt.nhs.uk. Within the following five working days, the local investigator may be required to provide additional information on the SAE in the form of a written narrative. This should include a copy of the completed SAE form, and any other diagnostic or relevant information that will assist the understanding of the event.

Additional and further requested information (follow-up or corrections to the original case) should also be added to eCRF using a new SAE Report Form. NHSBT CTU will ensure that all SAEs are reported to the sponsor.

The clinical reviewers will review the SAEs and, if they agree that the SAEs are unexpected and related, or pose an immediate risk to patient health or safety, then they will report them to the DMC immediately and to the device manufacturer and the REC within 15 calendar days of the chief investigator becoming aware of the event. The DMC will review the accumulating data at regular intervals.

### Frequency and plans for auditing trial conduct {23}

The investigators shall conduct this study in accordance with this protocol and any conditions of approval/notification imposed by the Research Ethics Committee and Competent Authority. Failure to comply with and/or inability to meet these regulations may jeopardise further participation of the investigator or investigative site in this and future clinical studies.

A ‘protocol deviation’ is a failure to adhere to the requirements specified in this study protocol without adequate justification. Examples may include the enrolment of a liver or recipient not meeting all of the inclusion/exclusion criteria specified in section 8 or missed study procedures without documentation. Livers excluded after randomisation due to factors not known at the time of randomisation (see procedures for reporting adverse events) will not be deemed protocol deviations.

#### Reporting of protocol deviations

All protocol deviations must be recorded and reported to the data monitoring committee. The DMC will review all deviations and assess their impact on patient safety. Serious breaches must be reported (see procedures for reporting serious breaches).

#### Reporting of serious breaches

A ‘serious breach’ is defined as a breach of GCP or the trial protocol which is likely to affect to a significant degree:

- The safety or physical or mental integrity of the subjects of the trial; or
- The scientific value of the trial

In the event that a serious breach is suspected, the NHSBT CTU must be contacted within one working day. In collaboration with the chief investigator and the DMC, the serious breach will be reviewed by the NHSBT CTU. If appropriate, NHSBT CTU, in conjunction with the sponsor, will report it to the REC committee and the host institution within seven calendar days.

### Plans for communicating important protocol amendments to relevant parties (e.g. trial participants, ethical committees) {25}

In the event of protocol amendments by the investigator, notification to the trial management group and NIHR (funder is required). In addition, the amendment documents and associated amendment tool is completed and sent to the sponsor for review and approval. Following sponsor approval, the amendment documents are submitted to the research ethics committee (REC) for authorisation. Amendments can only be implemented if a favourable opinion is granted by the REC.

## Dissemination plans {31a}

### Data analysis and release of results

By conducting the study, the local investigators agree that all information provided by the sponsor and co-ordinating centre will be maintained by the local investigators and the site personnel in strict confidence. It is understood that the confidential information provided to local investigators will not be disclosed to others without authorization from the sponsor and/or co-ordinating centre.

The scientific integrity of the study requires that all data must be analysed study-wide and reported as such. No data from the study will be presented in oral or written form without permission of the TSC. Approval to submit papers for publication will include all authors of the paper.

### Primary outcome publications

All publications, abstracts and other outputs will be reviewed by the trial steering committee (TSC) prior to publication. Publications will reflect the input of all participating centres in authorship, which will be agreed by the TSC.

Reports relating to primary outcomes will be published in peer-reviewed journals of appropriate relevance. Individual centres will undertake not to report any trial data independently. A final report on the primary outcomes of the study will be compiled by the chief investigator and NHSBT CTU and approved and signed off by each local investigator.

### Other study papers, abstracts and presentations

Study investigators wishing to publish secondary data analyses will submit a proposal to the TSC for approval. If the committee accepts the proposal, then the author of the proposal may decide on the lead in each publication resulting from such a proposal.

### Identification

The ISRCTN trial identifier will be included on all presentations and publications.

### Lay summary

Liver disease is the third leading cause of premature death in the UK. Liver transplantation is the only successful treatment for end-stage liver disease but is limited by a shortage of suitable donor organs. As a result, up to 20% of patients on the NHS liver transplant waiting list die before receiving a lifesaving transplant. However, a third of donated livers cannot be used for transplants. A frequent reason for this is the presence of fat within the liver cells (known as non-alcoholic fatty liver disease). This affects a third of the UK population and is commonest with obesity. As the incidence of obesity in the general population increases, donated organs are more likely to be fatty.

Transplanting a fatty liver carries a much greater risk to the patient compared to a normal liver. This is because fatty livers do not tolerate being cooled down, and we currently store organs in an ice box before the transplant. An alternative new technology (normothermic machine perfusion; NMP) stores the liver in very similar conditions to those in the body: it maintains the liver at body temperature and provides oxygen and nutrition. We know that this preserves it in better condition, with less damage to liver cells: it also allows the surgeon to test how well the organ is working before deciding whether to carry out the transplant. However, whilst beneficial, NMP technology does not completely resolve the problem of fatty livers, because fat remains in the liver cells.

We have been testing a new way to remove fat from the liver during NMP. We add a combination of (currently available) drugs to release fat from liver cells, and we remove the fat from the perfusion machine using a filter. This reduces the amount of fat in the liver and improves its function. None of the livers treated in this experimental study were actually transplanted: if used for patients, we believe that this might increase the number of livers that could be transplanted safely.

In the proposed trial, we will randomly assign 60 livers from donors with a high risk of fatty liver disease to either NMP alone or NMP with fat removal treatment. We will assess how many of these livers are safe to transplant and, in those that are transplanted, follow the outcomes after the operation. The main objective is to show whether this treatment is safe; it will also help us to design a future, larger study which will test the extent to which fat removal actually leads to additional transplants.

Patients and their families have contributed in the design of the study and will be members of the committee that run it. They believe that the study is addressing an important issue, particularly in the context of the global obesity crisis and its consequent implications for liver transplantation. They have concluded that this area of research is of great significance in order to reduce waiting list deaths.

## Discussion

This study addresses the paradox whereby patients die on the liver transplant waiting list whilst less than two thirds of deceased donors result in a transplanted liver in the UK. Poor utilisation of steatotic livers is exacerbated by the globally increasing incidence of obesity and the associated increase in frequency of hepatic steatosis in donors. If the benefit of NMP with ex-situ treatment of steatotic livers are confirmed, NHS practice will be influenced with much greater utilisation of moderately and severely steatotic organs. Evidence that supports this hypothesis is the primary purpose of this study.

More broadly, this research has the potential to move NMP into the realm of targeted interventions, i.e. ex-situ pharmacological optimisation of a donor organ prior to transplantation. However, there are number of considerations that will determine the success of this study.

### Potential barriers to research

#### Regulatory

The pharmacological agents that form an important part of the basis of this proposal, although readily available for patient use, are not licensed for this application. The MHRA has reviewed our application and advised that our proposal is not a clinical trial of an Investigational Medicinal Product (IMP) as defined by the EU Directive 2001/20/EC and no submission to the Clinical Trials Unit at the MHRA is required. This outcome has been in the context of a previous comparable negotiation between OrganOx Ltd. and the MHRA, over the use of sodium taurocholate. This is a choleretic agent, a bile salt of bovine origin, that is used to optimise biliary function in perfused livers and infused continuously throughout perfusion. The MHRA agreed that the flushing of the liver at the end of perfusion (with 2 L of preservation solution) resulted in below therapeutic levels of sodium taurocholate carried to the patient.

#### Cost

We are not proposing to carry out heath economic analysis as part of the proposed trial, but data collection is being planned in another NMP trial. In general, transplantation is highly cost-effective and it is expected that the cost of technology that increases the productivity of transplant units will fall below the NICE threshold of £20,000 per QALY.

#### Complexity

As noted above, all seven UK liver transplant centres have access to the OrganOx NMP system and have technical teams that are trained in its use. The additional skills to implement the defatting protocol, beyond those of NMP alone, will be well within the skill-set of the teams, with some specific extra training. NMP is increasingly used for steatotic livers, especially the moderately and severely steatotic organs that form the target for this technology, and so the additional complexity involved in adding the defatting protocol will be small. The evolution of objective criteria to determine transplantability will be a welcome benefit to clinical teams—this is a very difficult and high-stakes judgement which is currently made with limited information.

#### Priority

The perceived importance of this research is high: at the recent annual conference of the British Transplantation Society, a ‘Dragon’s Den’ session interrogated a number of proposed research initiatives, including NMP defatting. This was very highly rated in terms of both interest and importance. Utilisation of currently discarded organs is seen as probably the most pressing individual challenge in liver transplantation today. Indeed, patients are much more likely to die on the waiting list in the UK (as a result of the organ donor shortage) than in the year after their transplant.

### Adoption of technology

The powerful need to improve the utilisation of steatotic organs is recognised by patients (who are aware of the mortality rate on the waiting list, as well as the restrictions on waiting list access) and healthcare providers (both transplant teams and NHS Trusts), who are anxious to increase transplant rates for reasons of both patient benefit and unit efficiency. There are, therefore, both ‘push’ and ‘pull’ factors that will encourage the adoption of any new technology that enables safe transplantation of moderate/severely steatotic livers.

Adoption of technology of this sort attracts a high level of publicity within the wider public and media, mainly because of its obvious life-saving connotations. For this reason, it is likely to attract political attention. Much political support for organ donation and transplantation over the last 10 years has resulted in a greatly increased donation rate and the recent implementation of the ‘opting-out’ system of consent has further raised the profile of transplantation both politically and in the community.

The technical barriers to adoption are surmountable, particularly as liver NMP is now available in all seven UK liver transplant centres; the addition of defatting is technically well within the capabilities of clinical teams skilled in the operation of existing NMP. The underlying technology is CE marked; in January 2019, NICE approved the use of ex-situ machine perfusion for extracorporeal preservation livers for transplantation (with special arrangements for clinical governance).

There is a good track record in the implementation of novel techniques within transplantation in the UK. NHSBT (blood and transplant) is very supportive not only of translational research (such as the proposed study) but also of the adoption of novel therapies: indeed, one of the advisory groups to NHSBT, the ‘Research and Innovation Advisory Group’, was established with precisely this objective.

### Dissemination

#### Patients

We will work closely with patient groups (e.g. British Liver Trust) to ensure efficient dissemination of the results of our work to patients, carers and the general public. Two patient representatives on the project steering group will facilitate the dissemination of plain English summaries of the results to patient groups.

#### Wider public

Results will be disseminated via websites and social media channels. Our previous work has attracted interviews from the BBC and other television channels, national newspapers, the New Scientist and other national and international media. We will work closely with the media relations departments of the University of Oxford and the trial site hospitals to manage any interactions with the media. Information will be made available on websites of the University of Oxford and its relevant departments and also those of patient charities.

#### Professional colleagues

We will present our work to meetings of the British Transplantation Society, British Liver Transplant Group, British Association for the Study of the Liver, European Society of Organ Transplantation, American Society of Transplant Surgeons, Transplantation Society, International Liver Transplant Society and other national and international bodies. We will submit manuscripts describing the outcome of our research to high impact journals, both in specialist (transplant and hepatology) and general medical.

## NHS, government and policy makers

The 2014 report of the All-Party Parliamentary Hepatology Group made clear recommendations for NHS England to provide a strategy in addressing liver disease. The British Liver Trust estimates that the cost related to liver disease will reach £1 billion per annum in the next decade. It is certain that tackling liver disease is a governmental priority for reasons of patient welfare, healthcare burden and the societal implications. We will ensure that the results and potential benefits to NHS patients of this work are transmitted to NHS policy makers via a number of routes. These will include the Liver Advisory Group to NHSBT, annual commissioning meetings with NHS England (Highly Specialised Services) and the National Institute for Health and Care Excellence (NICE).

## Conclusion

This study explores ex-situ pharmacological optimisation of steatotic donor livers during NMP. If the intervention proves effective, it will allow the safe transplantation of livers that are currently very likely to be discarded (thereby reducing waiting list deaths) and provide data to inform the design a subsequent efficacy trial.

## Trial status

This is version 3.3 of the protocol, dated 1 March 2024. The DeFat study set-up tasks commenced in April 2021 and the study opened for recruitment on 23 February 2023. The anticipated end date for recruitment is on 30 November 2024 with 6-month follow-up of the last participant anticipated to be completed by 30 May 2025. The DeFat study is currently open for recruitment and it is anticipated that the primary outcome analysis report will be completed by 30 November 2025.

### Supplementary Information

Supplementary Material 1. 

Supplementary Material 2. 

## Data Availability

No data from the study will be presented in oral or written form without permission of the TSC. Approval to submit papers for publication will include all authors of the paper. Following publication of the trial results, reasonable data access request will be reviewed by the TSC.

## Abbreviations

°C: Degrees Celsius
%: Percent
3-OHB: 3-Hydroxybuturate
AE: Adverse event
APHG: All-Party Parliamentary Hepatology Group
AR: Adverse reaction
ALP: Alkaline phosphatase
ALT: Alanine transaminase
AST: Aspartate transaminase
BMI: Body mass index
CI: Chief investigator
CIT: Cold ischaemia time
CONSORT: Consolidated Standards of Reporting Trials
CRF: Case report form
CTA: Clinical trials authorisation
CTU: Clinical trials unit
DBD: Donation after brain death
DCD: Donation after circulatory death
DLI: Donor liver index
DMC/DMSC: Data monitoring committee/data monitoring and safety committee
DNL: De novo lipogenesis
DSUR: Development Safety Update Report
EAD: Early allograft dysfunction
ECD: Extended criteria donor
FGF-21: Fibroblast growth factor-21
FLI: Fatty liver index
GCP: Good clinical practice
GGT: Gamma-glutamyl transpeptidase
GW7647: 2-(4-(2-(1-Cyclohexanebutyl)-3-cyclohexylureido)ethyl)­phenyl­thio)-2-methyl­propionic acid
GW501516: 2-(2-Methyl-4-(((4-methyl-2-(4-(trifluoromethyl)phenyl)-5-thiazolyl)methyl)thio)phenoxy)-acetic acid
HBV: Hepatitis B virus
HCV: Hepatitis C virus
HD: Haemodialysis
HDF: Haemodiafiltration
HIV: Human immunodeficiency virus
HF: Haemofiltration
HRA: Health Research Authority
IB: Investigators Brochure
ICF: Informed consent form
IFU: Instructions for use
IHTG: Intrahepatocellular triglyceride
INR: International normalised ratio
IP: Intellectual property
IRI: Ischaemia-reperfusion injury
IRB: Independent Review Board
ITU: Intensive care unit
IVC: Inferior vena cava
kg/m^2^: Kilogramme per square metre
LD: Lipid droplet
MAP: Mean arterial pressure
MaS: Macrovesicular steatosis
MEAF: Model of Early Allograft Function
MELD: Model for end-stage liver disease
MHRA: Medicines and Healthcare products Regulatory Agency
MRI: Magnetic resonance imaging
NAFLD: Non-alcoholic fatty liver disease
NICE: National Institute for Health and Care Excellence
NHS: National Health Service
NHSBT: NHS Blood and Transplant
NMP: Normothermic machine preservation
RES: Research Ethics Service
RGEA: Research Governance, Ethics and Assurance, University of Oxford
PI: Principal investigator
PIL: Participant/patient information leaflet
PNF: Primary non-function
PPARα: Peroxisome proliferator-activated receptor alpha
PPARδ: Peroxisome proliferator-activated receptor delta
PPI: Patient and public involvement
PRISMA: Preferred Reporting Systematic Reviews and Meta‐Analyses
PRS: Post reperfusion syndrome
R&D: NHS Trust R&D Department
REC: Research ethics committee
RNA: Ribonucleic acid
RRT: Renal replacement therapy
RSI: Reference Safety Information
SADE: Serious adverse device effect
SAE: Serious adverse event
SAP: Statistical analysis plan
SAR: Serious adverse reaction
SCD: Standard criteria donor
SCS: Static cold storage
SDV: Source data verification
SMPC: Summary of Medicinal Product Characteristics
SNOD: Specialist Nurses in Organ Donation
SOP: Standard operating procedure
SUSAR: Suspected unexpected serious adverse reactions
TG: Triacylglycerol
TMF: Trial Master File
TMG: Trial Management Group
TPN: Total parenteral nutrition
TSC: Trial Steering Committee
UKCRC: UK Clinical Research Collaboration
UKTR: UK Transplant Registry
USADE: Unanticipated serious adverse device event
UW: University of Wisconsin
VLDL: Very low-density lipoprotein
