# Supplementary material for: Defatting of donor transplant livers during normothermic perfusion—a randomised clinical trial: study protocol for the DeFat study
Source: Trials. 2024 Jun 17;25:386. doi: 10.1186/s13063-024-08189-4 (PMC11181618; doi:10.1186/s13063-024-08189-4)
Supplement: Supplementary file 1 — Supplementary Material 1. [file 13063_2024_8189_MOESM1_ESM.docx]

**Supplemental Table 1.** Clavien-Dindo classification of surgical complications (66)

| **Grade** | **Definition** |
| --- | --- |
| **I** | Any deviation from the normal postoperative course without the need for pharmacological treatment or surgical, endoscopic and radiological interventions. |
| **II** | Requiring pharmacological treatment with drugs other than such allowed for grade I complications. Blood transfusions and total parenteral nutrition are also included. |
| **III** | Requiring surgical, endoscopic or radiological intervention. |
| **IIIa** | Intervention not under general anaesthesia. |
| **IIIb** | Intervention under general anaesthesia. |
| **IV** | Life-threatening complications (including CNS complications) requiring HDU/ITU management. |
| **IVa** | Single organ dysfunction (including dialysis). |
| **IVb** | Multi-organ dysfunction. |
| **V** | Death of a patient. |
| **Suffix ‘d’** | If the patient suffers from a complication at the time of discharge, the suffix ‘d’ (for disability) is added to the respective grade of complication. This label indicates the need for a follow-up to fully evaluate the complication. |
