# Supplementary material for: Defatting of donor transplant livers during normothermic perfusion—a randomised clinical trial: study protocol for the DeFat study
Source: Trials. 2024 Jun 17;25:386. doi: 10.1186/s13063-024-08189-4 (PMC11181618; doi:10.1186/s13063-024-08189-4)
Supplement: Supplementary file 2 — Supplementary Material 2. [file 13063_2024_8189_MOESM2_ESM.zip › DeFat_Consent_v1.2_2023_06_26_Final_Clean_ESM.docx]

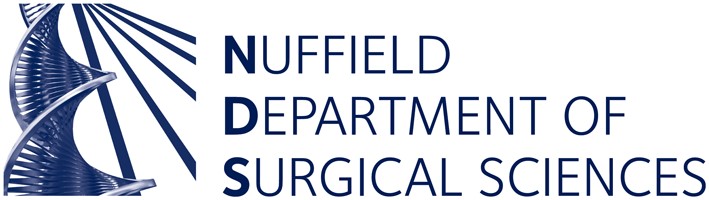

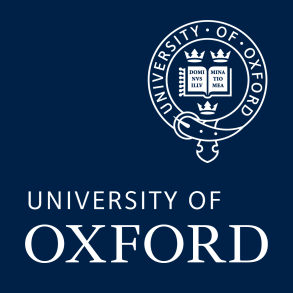


Chief Investigator: Professor Peter Friend

Operational Lead: Mr Simon Knight

Trial co-ordinator: Mr Hussain Abbas

Oxford Transplant Centre

Churchill Hospital

Old Road, Headington

Oxford, OX3 7LE

*Site ID Code: Participant identification number:*

**R**

**CONSENT FORM**

**The DeFat study**

Defatting of donor transplant livers during normothermic perfusion – a randomised clinical trial

*If you agree, please initial the box*

| 1. I confirm that I have read the information sheet dated.................... (version............) for this study. I have had the opportunity to consider the information, ask questions and have had these answered satisfactorily. |  | |
| --- | --- | --- |
| 1. I understand that my participation is voluntary and that I am free to withdraw at any time without giving any reason, without my medical care or legal rights being affected. |  | |
| 1. I understand that relevant sections of my medical notes and data collected during the study may be looked at by individuals from University of Oxford, from regulatory authorities and from the participating NHS Trust(s), where it is relevant to my taking part in this research. I give permission for these individuals to have access to my records. |  | |
| 1. I understand that the information held and maintained by the NHS Blood and Transplant Registry and my local transplant centre may be used to help contact me or provide information about my health status after the end of this study in order to assess longer-term outcomes. |  | |
| 1. I understand that if I withdraw from the study at any time, data collected to that point will continue to be used in this research. |  | |
| 1. I understand and agree that my samples will be used in research aimed at understanding the genetic influences on disease and that the results of these investigations are unlikely to have any implications for me personally. |  | |
| 1. I understand that the MRI scan will be a research scan. If a concern is raised about a possible abnormality on my scan, I will only be informed if a doctor thinks it is medically important such that the finding has clear implications for my current or future health. |  | |
| 1. I agree for my anonymised samples to be used in future research, here or abroad, which has ethics approval. I understand this research may involve commercial organisations. |  | |
| 1. I agree to take part in this study. |  | |
| 1. I agree to be contacted about potential participation in future research | Yes | No |

| *_______________________* | *_________________* | *___________________________* |
| --- | --- | --- |
| *Name of Participant* | *Date and time (hh:mm)* | *Signature* |
| *_______________________* | *_________________* | *___________________________* |
| *Name of Person taking Consent* | *Date* | *Signature* |

| Was consent taken on the telephone? | Yes | No |
| --- | --- | --- |

When completed: 1 for participant; 1 for researcher site file (original); 1 to be kept in medical notes
