# Supplementary material for: Defatting of donor transplant livers during normothermic perfusion—a randomised clinical trial: study protocol for the DeFat study
Source: Trials. 2024 Jun 17;25:386. doi: 10.1186/s13063-024-08189-4 (PMC11181618; doi:10.1186/s13063-024-08189-4)
Supplement: Supplementary file 2 — Supplementary Material 2. [file 13063_2024_8189_MOESM2_ESM.zip › DeFat_PIS_v1.3_2023_06_26_Final_Clean_ESM.docx]

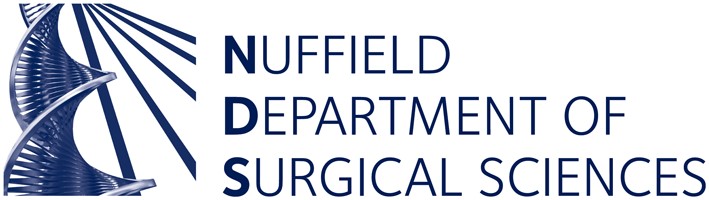

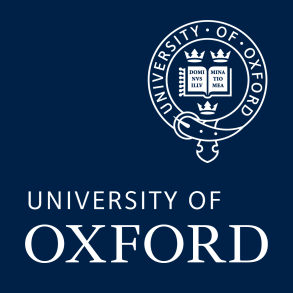


Chief Investigator: Professor Peter Friend

Operational Lead: Mr Simon Knight

Trial co-ordinator: Mr Hussain Abbas

Oxford Transplant Centre

Churchill Hospital

Old Road, Headington

Oxford, OX3 7LE

**PARTICIPANT INFORMATION SHEET**

**The DeFat study**

**Defatting of donor transplant livers during normothermic perfusion – a randomised clinical trial**

We'd like to invite you to take part in our research study. Before you decide, it is important that you understand why the research is being done and what it would involve for you. Please take time to read this information and discuss it with others if you wish. If there is anything that is not clear, or if you would like more information, please ask us.

# Summary – Key Information

We would like to assess whether a new technique for removing fat from a liver can improve the safe and successful use of livers that are ordinarily offered for transplant but are retrieved from donors at risk of having a fatty liver. Such livers do not tolerate being cooled during conventional cold storage in an ice-box and are at risk of injury during the transplant procedure. The objective of this study is to make fatty livers safer for transplantation and thereby increase the number of transplants that can be safely conducted.

This new technique is based on technology, called normothermic machine perfusion, that maintains the liver in a functioning state outside the body on a machine, providing oxygen and nutrition at a normal body temperature. There are several benefits:

1. Recovery from injury sustained prior to, during organ retrieval and following cold storage
2. Assessment of organ function prior to transplantation: allowing livers (even those with higher fat content) that are functioning on the machine to be transplanted safely
3. Extended preservation times (up to 24 hours)
4. The opportunity for therapeutic intervention (removal of fat) to a functioning organ before it is transplanted

It is important to understand, that these livers are not discarded organs: these are donor livers that have been retrieved for the intent of transplantation. You would have been offered this liver whether, or not you agree to take part in the study. Such livers are offered and used for transplant every day in the UK, and your transplant surgeon will decide whether to accept an offer based upon the donor’s details, your needs, and a thorough assessment of the liver on arrival at your transplant centre.

Taking part in this study does not increase your chances of an organ offer – your place on the waiting list will not change.

The aim of the study is to demonstrate whether the fat removal process is safe. There are no anticipated side effects or risks related to the preservation and defatting process itself on the machine. Your new liver will be randomly allocated to receive either normothermic perfusion alone or normothermic perfusion with the additional process of fat removal treatment (defatting) in order to test whether liver function improves prior to transplantation.

This information will help your surgeon to decide whether the liver is suitable for transplantation based on how well it is functioning on the machine. If there are any concerns about the liver function during preservation, the liver will not be transplanted. This decision will be made after carrying out a functional assessment of the liver following a minimum preservation period of 6 hours on the machine. If the transplant proceeds, you will be monitored closely with regular observation and tests as per the standard of care for liver transplantation.

# What is the purpose of the study?

# *Background*

Liver disease is the third leading cause of premature death in the UK. Liver transplantation is the only successful treatment for end-stage liver disease but is limited by a shortage of suitable donor organs. As a result, up to 20% of patients on the NHS liver transplant waiting list die before receiving a lifesaving transplant.

However, a third of donated livers cannot be used for transplants. A frequent reason for this is the presence of fat within the liver cells (known as non-alcoholic fatty liver disease). This affects a third of the UK population and is commonest with obesity. As the incidence of obesity in the general population increases, donated organs are more likely to be fatty.

Transplanting a fatty liver carries a greater risk to the patient compared to a normal liver. This is because fatty livers do not tolerate being cooled down, and we currently store organs in an ice-box before the transplant. An alternative new technology, called normothermic machine perfusion (NMP) stores the liver in very similar conditions to those in the body: it maintains the liver at body temperature and provides oxygen and nutrition. Evidence from large multicentre clinical trials in the United Kingdom, Europe and North America in the past decade confirm that NMP preserves the liver in a better condition, with less damage to liver cells: it also allows the surgeon to test how well the organ is working before deciding whether to carry out the transplant.

Whilst beneficial, NMP technology does not completely resolve the problem of fatty livers because fat remains in the liver cells. During early pre-clinical experiments by our group on fatty pig livers, release of fat from the liver was noted during NMP. However, the fat remained in the circuit during perfusion thereby limiting the amount that could be extracted by NMP alone.

This finding was also confirmed during the analysis of a cohort of fatty livers that were preserved using NMP alone and then transplanted as part of a large clinical trial. When biopsies were examined under the microscope, the amount of fat in the liver cells appeared unchanged over the preservation period. This meant that these livers were still susceptible to greater injury (demonstrated on blood tests) following the transplant operation compared to normal livers.

To address this problem, in a previous study on discarded human livers we successfully tested a new way to remove fat from the liver during NMP. We added a combination of drugs to release fat from liver cells, and we removed the fat from the perfusion machine using a filter. This reduced the amount of fat in the liver and improved its function. None of the livers treated in this experimental study were actually transplanted. However, if used for patients, we believe that this might increase the number of livers that could be transplanted safely due to improved preservation, reduction in fat content and improved tolerance to injury during and following the transplant operation.

*This study*

In this clinical trial, we will randomly assign 60 livers from donors with a high risk of fatty liver disease to either NMP alone or NMP with fat removal treatment. We aim to assess how many of these livers are safe to transplant and, in those that are transplanted, follow the outcomes after the operation. The main objective is to show whether this treatment is safe. In addition, it will also help us to design a future, larger study which will test the extent to which fat removal actually leads to additional transplants.

Patients and their families have contributed to the design of this study and will be members of the committee that run it. They believe that the study is addressing an important issue, particularly in the context of the global obesity crisis and its consequent implications for liver transplantation. They have concluded that this area of research is of great significance in order to reduce waiting list deaths.

We plan to present the results of this 3-year project at national and international conferences and publish this research in high-impact journals. This will ensure that transplant teams around the world become aware of this treatment.

# Why have I been invited?

# You have been invited to take part in this study because you are on the liver transplant waiting list in a UK transplant centre and could be allocated a liver that is less likely to result in a transplant (due to presence of fat) and meets the criteria for the study. You will have had the opportunity to discuss study enrolment in clinic or on the telephone. If you wish to participate in the study, you will have the option of signing the informed consent form whilst on the waiting list or once admitted to hospital for your liver transplant.

# Do I have to take part?

# No. It is up to you to decide whether you take part in the study. If you decide to take part, you are free to change your mind at any time and without giving a reason.

If your transplant surgeon feels that the liver allocated to you is suitable to transplant, and you are happy to proceed, you will receive this transplant, whether or not you agree to take part in the study. Your chances of a transplant and the care you receive are not affected by whether you agree to take part in the study or not. If you decide not to take part in the study, no further information will be collected about you and your information will not be used for the study.

# What will happen to me if I decide to take part?

The liver that will be allocated to you will be preserved either using the NMP with fat removal treatment, or NMP alone. This will be decided randomly by a computer.

Prior to randomisation your transplant surgeon will use clinical information about the donor and the appearance of the liver to make a decision as to whether the liver is suitable for inclusion in the study (due to risk of containing excess fat). If the liver is accepted, it will be stored on the machine (with or without defatting treatment) and your surgeon will use all of the information available to help to make the decision if the liver is safe to transplant. Neither you or your surgeon will know if the liver has undergone the defatting treatment or not.

Occasionally, the donor liver may arrive at the hospital before you. There is a chance that randomisation and perfusion of the donor liver may commence prior to your arrival at hospital. This will only happen if you have already signed the consent form whilst on the waiting list. In this scenario, you will be contacted by phone prior to randomisation to reaffirm consent. If you choose to withdraw, the liver will not be randomised and will be excluded from the study and offered as per standard care.

If your transplant goes ahead, we will collect information about your operation and the outcomes from your transplant when you attend routine hospital appointments for 6 months after your transplant. This includes information during the transplant operation, during your stay in hospital, and at clinic appointments at 1 month, 3 months and 6 months after your transplant.

Below is a summary of the data that we will collect during the study, if your transplant goes ahead:

*At the time of your transplant*

At the time of your transplant, we will collect information about:

- You – your age, gender, weight, height, body mass index, waist circumference, medical history and cause of liver failure
- Your donor – their age, gender, weight, height, body mass index, waist circumference, medical history, blood tests and cause of death
- Your transplant – how the operation is done, timings, blood transfusions and other treatments
- Additional research blood tests will also be taken and will align with your routine blood sample collection.

*During your time in hospital*

Whilst you are in hospital, we will collect information about your progress every day. This will include:

- How well your transplant is working
- Blood tests including liver and kidney function. These are routine tests performed in hospital for all patients. Additional research blood tests will also be taken and will align with your routine blood sample collection.
- Need for dialysis
- Any complications, scans or additional tests
- The medications that you are taking

*After your transplant*

Once you have been discharged, we will see you during a normal clinic visit 1, 3 and 6 months after your transplant. We will look at your progress, including:

- How well your transplant is working
- Blood tests including liver and kidney function. These are routine tests performed in hospital for all patients. Additional research blood tests will also be taken and will align with your routine blood sample collection.
- Need for dialysis
- Any hospital visits, complications, scans or additional tests
- The medications that you are taking

*Longer-term monitoring*

We would like to see what happens to your transplant in the longer-term (1 year). All transplant recipients in the UK take part in the UK Transplant Registry, which stores information about the health of your transplant and any problems. We will ask for your permission to use the information in this registry to check your health status after one year and any missing information. Only authorised staff at NHSBT will have access to this information and it will not be shared externally.

**Research procedures**

Very small samples (biopsies) of the transplant liver will be taken - before being placed for storage on the machine, after storage, and just before the end of your transplant operation (described in detail on page 6). The end of the bile duct (the tube that drains your new liver) will also be trimmed at each of these time points to provide small research samples. These samples will only be taken if there is sufficient length on the bile duct and it is safe and feasible to do so.

Research blood samples (8-10mls) will also be taken during your operation, whilst you are an inpatient, during your follow-up visits (month 1, 3 and 6) and will align with your routine blood tests to avoid any extra visits. These research biopsy and blood samples are taken to assess how healthy the liver is over the storage time and following your operation. We will also arrange an MRI scan of your liver to coincide with your 6-month clinic appointment (described in detail on page 7). The purpose of this research scan is to evaluate the overall health of your liver following your operation.

**Standard care procedures**

The routine samples taken during this study (whilst an inpatient and during follow-up in clinic) are part of your normal clinical care. These tests will determine how well your new liver is functioning, and would be taken routinely even if you were not participating in the trial. They will be processed in the laboratories in your hospital in the usual way and the results made available to your doctors to help them to look after you.

# What should I consider?

You may not be able to take part in this study if:

- Your donor is less than 18 years of age
- The liver that you are being offered is not considered at high-risk of being fatty
- You are having another organ transplanted as well as the liver
- You have rapid onset, severe liver injury with failure of liver function (acute/fulminant liver failure)
- Your transplant centre cannot offer machine perfusion due to machine or staff availability
- The liver allocated to you is being split to allow two transplants
- You have a contra-indication to MRI e.g. pacemaker (see page 7)
- Your medical team feels for any reason you are not suitable to take part. Your medical team will discuss this with you.

Your care at the time of transplant, and afterwards, does not differ from normal care in your transplant centre. All of your medications and tests/treatments will stay the same other than research blood tests taken during and after your operation as well as a MRI scan of your liver which will coincide with your 6 month clinic appointment. These research tests will align with samples taken as part of your routine clinical care whilst you are inpatient and in outpatient follow-up visits.

Your chances of being offered a liver for transplant are not affected by taking part in the study – it will neither increase nor decrease your chances of an offer.

If you have been asked to take part in this study, it is because you have been offered a liver that is less likely to result in a transplant (due to a high-risk of being fatty). It is important to understand that you would have been offered this liver anyway, and that your transplant surgeon would have considered the risks and benefits before deciding whether to go ahead with the transplant.

If you decide not to take part, you will still receive the liver allocated to you if it is suitable for transplant. If you are taking part in another research study, please let the person discussing this study with you know. It may still be possible for you to take part if your team feel that the studies will not affect one another.

# Are there any possible disadvantages or risks from taking part?

Normothermic machine preservation (NMP) has already been shown to be safe in large clinical trials. In these studies, patients did at least as well on average when the liver underwent machine perfusion, and there was no increase in complications. NMP has subsequently been used in over 1500 transplants worldwide and is in use in all seven UK liver transplant centres.

In addition, we have been testing a new way to remove fat from the liver during NMP. We add a combination of drugs to release fat from liver cells, and we remove the fat from the perfusion machine using a filter. This reduces the amount of fat in the liver and improves its function on the machine. None of the livers treated in this experimental study were actually transplanted: if used for patients, we believe that this might increase the number of livers that could be transplanted safely. The drugs (targeting fat metabolism) and filter (for removal of excess circulating fat) are used in other healthcare settings. All drugs will be flushed out of the liver prior to transplantation.

The liver allocated to you will only be transplanted if your transplant surgeon feels that it is safe to do so, given all of the information available.

*Liver Biopsy*

A liver biopsy is a medical test where a small sample of tissue is retrieved from the liver using a needle to be examined under a microscope. A number of very small biopsies of the transplant liver will be taken - before being placed for storage on the machine, after storage, and just before the end of your transplant operation. These samples will be taken under direct vision and will not affect the function of the liver. We will use the biopsies to assess for liver fat content, injury and perform genetic analysis in order to identify genes that contribute to liver fat accumulation in the donor at a later date. Overall, we will be able to use this information to determine how healthy the liver is over the storage time.

*MRI Scan*

An MRI scan is a safe and non-invasive scan and does not involve any ionising radiation (x-rays). However, because MRI uses a large magnet to work, MRI scans are not suitable for everybody. Because of this, you will be asked pre-screening safety questions to help determine if you are able to take part. For example, if you suffer from claustrophobia, you could not be scanned. Also, MRI scanning for research purposes would not be performed without further investigation if you have a:

- Heart pacemaker
- Mechanical heart valve
- Mechanical implant such as an aneurysm clip
- Hip replacement, or if you carry other pieces of metal that have accidentally entered your body

While there is no evidence to suggest that MRI is harmful to unborn babies, as a precaution, the Department of Health advises against scanning pregnant women unless there is a clinical benefit.  We do not test for pregnancy as routine so if you think you may be pregnant you should not take part in this study. As some of the scans are noisy, you will be given earplugs, head padding or headphones to make this quieter for you. It is important that these are fitted correctly as they are designed to protect your ears. In preparation for your scan and for your comfort and safety we may ask you to change into pocketless and metal free "pyjama-style" top and trousers, which are available in a range of sizes. You may keep your underwear and socks on, but we would ask that items such as underwired bras and jewellery (including body piercings) must be removed. If you have a suitable sports type bra you may use this instead. Some tattoos, eye shadow and mascara must also be avoided since some types contain materials that can interact with the magnetic field. If you have a tattoo, you will be asked to tell the radiographer immediately if you experience any discomfort or heat in your tattoo during the scan. If you wish to wear eye makeup to your scan we can provide makeup removal wipes but you are advised to bring your own makeup to reapply. Lockers are provided to secure your personal belongings and clothing.

Some people scanned in MRI scanners (especially newer ultra-high resolution machines), experience a mild dizzy sensation as they are moved into the scanner. This is normal and the sensation starts to go away once in the scanner.

It is important to note that this is **not** an MRI scan for diagnostic purposes, and therefore these scans are not a substitute for a doctor’s appointment. The MRI will be reported and the results will be available to the local transplant teams. However, the scans are **not** routinely looked at by a doctor; rather the scans are intended for research purposes only. Occasionally a possible abnormality may be detected. In this case, we would have the scan checked by a doctor. If the doctor felt that the abnormality was medically important, you would be contacted directly and recommended to have a hospital (NHS) diagnostic scan arranged. All information about you is kept strictly confidential.

# What are the possible benefits of taking part?

Taking part in this study does not increase your chances of an organ offer – your place on the waiting list will not change.

As your liver will undergo normothermic machine perfusion alone or with fat removal treatment, it is possible that the additional information from the device may improve the confidence of your surgeons in deciding whether to transplant the liver, and/or improve the condition of the liver. However, the reason that we are undertaking this study is that the effect of defatting on post-transplant outcomes is uncertain, so no benefit can be promised. The study may help us understand how we can increase the availability of donor organs and may benefit other people in the future.

# Will my General Practitioner/family doctor (GP) be informed of my participation?

# As the method of organ preservation does not affect your long-term care, your GP will not routinely be informed about your participation in this study. However, we are happy to provide information if required.

# Will I be reimbursed for taking part?

You will not receive any payment if you agree to take part in this study.

Where possible, your study visits will take place at routine outpatient appointments to avoid and additional visits to hospital. On the rare occasions where an additional visit is required, we will pay reasonable travel expenses upon production of receipts.

# What will happen to the samples I give?

Several very small biopsies of the transplant liver will be taken - before being placed for storage on the machine, after storage, and just before the end of your transplant operation. A bile duct biopsy will also be taken at each of these time points (providing there is sufficient length on the bile duct, and it is feasible to do so). These research samples will be transported to Oxford University Hospitals for storage and subsequent processing.

Research blood samples will be collected during your operation and will be transported to Oxford University Hospitals for storage and subsequent processing. Post-operative blood samples will also be taken whilst you are an inpatient and during your outpatient follow-up visits. The collection of these research samples will align with your routine blood tests in order to avoid any additional hospital visits and will be sent to specialist accredited laboratories for processing in the United Kingdom (UK) or abroad.

All research samples will be stored for future research and mechanistic studies described in the study protocol. As we have requested consent for use of samples for future research you will not be asked to reconsent at any stage.

The majority of the blood samples to be taken during this study are part of your normal clinical care. These will be processed in the laboratories in your hospital in the usual way and the results made available to your doctors to help them to look after you. These tests will determine how well your new liver is functioning, and would be taken routinely even if you were not participating in the trial. Usually, these will be taken following your transplant (whilst you are an inpatient) and at your follow-up visits at approximately 30 days, 3 months and 6 months after liver transplant. Any residual samples will be discarded as per routine hospital procedure.

# Will my taking part in the study be kept confidential?

# Your participation in this study will be kept confidential. Data will be collected and stored in an electronic research database. Your data will be de-identified and stored in the database by a unique study code (Trial ID). Information to identify you from your study code will be stored securely at your own transplant centre, to allow anonymity to be broken if needed for safety purposes. Your consent to participate in the study will also be stored securely at your own centre.

# The research database will be stored securely under password protection on a central NHS Blood and Transplant (NHSBT) server. Only staff involved in the study and who have received adequate training will have access to the database.

# Responsible members of the University of Oxford, NHS Blood and Transplant and participating NHS trusts may be given access to data for monitoring and/or audit of the study to ensure that the research is complying with applicable regulations.

# When we publish the results of this trial the data that is reported will be a summary of all patients that take part. It will not be possible to identify individual patients from the data which is published.

# What will happen to my data?

Our procedures for the handling, storage and destruction of the data are compliant with the UK General Data Protection Regulation (GDPR) and the Data Protection Act 2018. These regulations require that we state the legal basis for processing information about you. In the case of research, this is ‘a task in the public interest.’ The University of Oxford, based in the United Kingdom is the data controller and is responsible for looking after your information and using it properly.

During enrolment you will be assigned a unique number (Trial ID) that will be used instead of your personal identifiers thereafter, this process is called pseudonymisation. Your personal data will be automatically linked to this number to ensure that statisticians and data managers in NHSBT can still link your information from the various sources, but without being able to identify you during the statistical analysis. In a similar way your research blood and biopsy samples will be labelled with this number to avoid sharing any of your personal details with the external laboratories but at the same time keep your samples linked to you. Sealed Envelope will use non identifiable information to assign this unique trial ID number during the process of randomisation.

We will be using information from you and your medical records and the NHS Blood and Transplant Registry in order to undertake this study and will use the minimum personally identifiable information possible. We will keep identifiable information about you for up to 5 years after the study has finished.

The [local NHS trust] will use your name, NHS number, address and contact details to send you information about this study and to oversee the quality of the study. They will also use it to contact you about the results of the study, and about any future research studies that you may be eligible for if you consent to this. If you agree to your details being held in order to be contactable regarding future research, the local trust will retain a copy of your consent form until such time as your details are removed from our database, keeping the consent form and your other details separate. All contact will come from your research team in the first instance. Agreeing to be contacted does not oblige you to take part in future research.

Before the statistical analysis is carried out, all your data will be gathered in a secure database on a secure platform within NHSBT. The results of the study will be reported and published in summary tables and graphs which means your identity and participation will be kept confidential.

Certain authorised individuals from the NHSBT Clinical Trials Unit or Research Governance, Ethics & Assurance Team (RGEA) at the University of Oxford might be given access (either locally or remotely) to the consent form and information from your medical record to assess the quality of the study in which case your personal information will be visible to them. Also, we may have to use your details to investigate or report a complaint.

UK GDPR provides you with control over your personal data and how it is used. When you agree to your information being used in research, however, some of those rights may be limited in order for the research to be reliable and accurate. Further information about your rights with respect to your personal data is available at:

<https://compliance.web.ox.ac.uk/individual-rights>

<https://www.hra.nhs.uk/information-about-patients/>

You can find out more about how we use your information by contacting [CI or study team email]

If you wish to obtain copy of the information held by NHSBT or the NHS Trusts you should contact the Trial Management team by sending an email to [DeFat@nhsbt.nhs.uk](mailto:DeFat@nhsbt.nhs.uk), you will be provided with further guidance.

# [What will happen if I don't want to carry on with the study?](http://hra-decisiontools.org.uk/consent/content-sheet-support.html#two)

Participation in this study is voluntary and you are free to withdraw at any time without providing a reason. If you decide not to continue with the study you will not be required to attend any further study visits, and no further data will be collected for the study. From the point of withdrawal from the study, no further information relating to you or your outcomes following your transplant operation will be accessed from the NHS Blood and Transplant registry.

Should you decide to withdraw from the study, your medical care will not be affected. You will continue to receive the care offered to all transplant recipients at your centre.

# Loss of capacity

The risk of a permanent loss in capacity (for example, due to a stroke) following a liver transplantation is very low. In the event of a prolonged loss of capacity to consent to continued involvement in the trial, we would provide your designated next of kin/consultee with information about the study (as described in the Patient Information Sheet).

We would seek advice from your designated next of kin/consultee about your continued participation in the study including collection of samples and data whilst your capacity is impaired in this manner. They would be provided with a consultee information pack and asked to sign a consultee declaration form regarding your continued involvement in the study. Both you and your designated consultee will receive a copy of the declaration form if/when you regain capacity.

# What will happen to the results of this study?

If the intervention proves effective, it will allow the safe transplantation of livers that are currently very likely to be discarded: this will increase the number of transplants that can be performed safely and have the effect of reducing waiting list deaths. The results of the trial will be presented in a way that masks your identity (summary tables, graphs) and disseminated via high-impact publications, presentation at conference presentations and press releases. We are also required to provide a report to the funder of the study (the National Institute of Health Research; NIHR).

We will work with patient groups to ensure efficient dissemination of our results to patients, carers and the general public. These will also be shared with NHS bodies and decision makers including NICE, NHSBT and NHS England.

This research being undertaken will also contribute to the fulfilment of a doctoral thesis of trainee surgeon Mr Syed Hussain Abbas, who is being supervised by Professor Peter Friend and Professor Leanne Hodson at the University of Oxford.

Your data will not be used to sell anything. It will not be given to other organisations or companies except for research. No data will be passed to any third parties and commercial entities other than for academic research collaboration related to this study.

We plan to present the results to patient groups to let them know what we have found. We will also make the results of the study available on the study website.

Your personal details will not be included in any of these reports – all data will be combined together and reported anonymously.

# What if we find something unexpected?

# We do not anticipate that there will be any unexpected clinical findings as a result of this study, as most of the data collected from you will form part of your routine clinical care. Should we find anything unexpected (for example on MRI scan or genetic analysis of biopsy samples from the donor liver) it will be communicated to your local transplant team so that they can ensure that any action required is taken.

# What if there is a problem?

The University of Oxford, as Sponsor, has appropriate insurance in place in the unlikely event that you suffer any harm as a direct consequence of your participation in this study. NHS indemnity operates in respect of the clinical treatment which is provided.

If you wish to complain about any aspect of the way in which you have been approached or treated, or how your information is handled during the course of this study, you should contact <name of local investigator><contact details (phone number & email)> or you may contact the University of Oxford Research Governance, Ethics & Assurance (RGEA) office on 01865 616480, or the head of RGEA, email [rgea.complaints@admin.ox.ac.uk](mailto:rgea.complaints@admin.ox.ac.uk).

The Patient Advisory Liaison Service (PALS) is a confidential NHS service that can provide you with support for any complaints or queries you may have regarding the care you receive as an NHS patient. PALS is unable to provide information about this research study.

If you wish to contact the PALS team, please contact <insert relevant NHS site phone number and email from the PALS website>.

If you are not happy with their response or believe they are processing your data in a way that is not right or lawful, you can complain to the Information Commissioner’s Office (ICO) ([www.ico.org.uk](https://ico.org.uk/)  or 0303 123 1113).

# How have patients and the public been involved in this study?

Previous liver transplant patients and their families were involved in the design of this study. They commented on the frequency of visits and tests, and the consent processes for the study.

The study team includes a previous liver transplant recipient, who has helped in the design of patient information and consent materials. The patient information and consent materials were also reviewed by the NHS Blood and Transplant Public Advisory Group.

# Who is organising and funding the study?

This study is being organised by researchers at the University of Oxford, in collaboration with the transplant team in your centre. The study is funded by the National Institute for Health Research (NIHR) and sponsored by the University of Oxford. The NHS Blood and Transplant Clinical Trials Unit will manage this study.

Your hospital will receive funding to cover the costs of including you in the study. Individual doctors or researchers will not be paid directly.

# Who has reviewed the study?

All research in the NHS is looked at by an independent group of people, called a Research Ethics Committee, to protect participants’ interests. This study has been reviewed and given favourable opinion by _______________Research Ethics Committee.

# Further information and contact details:

[Please](mailto:Please) contact < > local research team < >(telephone, e-mail, in writing)

*Thank you for considering taking part. If you agree to take part, you will be given a copy of this information sheet and a signed copy of your consent form to keep.*
